# Emerging frontiers in SERS-integrated optical waveguides: advancing portable and ultra-sensitive detection for trace liquid analysis

**DOI:** 10.1038/s41377-025-01989-6

**Published:** 2025-11-26

**Authors:** Danheng Gao, Jiahao Liu, Xiao Liu, Kang He, Zhanyu Ma, Huan Liu, Jihou Wang, Qihan Zhang, Zhaonan Huang, Meng Luo, Haoran Meng, Rui Du, Juntao Gao, Qing Wu, Xinghua Yang

**Affiliations:** 1 State Key Laboratory of Advanced Manufacturing for Optical Systems, Changchun Institute of Optics, Fine Mechanics and Physics, Chinese Academy of Sciences, Changchun, 130033 China; 2 Division of Thyroid Surgery, The China-Japan Union Hospital of Jilin University, Jilin Provincial Key Laboratory of Surgical Translational Medicine, Jilin Provincial Precision Medicine Laboratory of Molecular Biology and Translational Medicine on Differentiated Thyroid Carcinoma, Changchun, 130033 China; 3 School of Basic Medical Sciences Tsinghua University Biomedicine Hall, Beijing, 100084 China; 4 Heilongjiang Province Key Laboratory of Laser Spectroscopy Technology and Application, Harbin University of Science and Technology, Harbin, 150080 China; 5 Key Laboratory of In-Fiber Integrated Optics, Ministry of Education, College of Science, Harbin Engineering University, Harbin, 150001 China

**Keywords:** Raman spectroscopy, Optofluidics

## Abstract

Surface-Enhanced Raman Scattering (SERS) integrated with optical waveguide sensing offers a transformative approach to overcoming the limitations of conventional SERS techniques, such as complex alignment requirements and limited signal collection efficiency. By leveraging the unique properties of optical waveguides, this integration significantly enhances detection sensitivity, simplifies sensor design, and enables the analysis of ultra-low concentration analytes in trace-volume samples. This review explores the latest advancements in combining diverse optical waveguide architectures with SERS technology, focusing on strategies to optimize the sensing interface and SERS substrate design for maximal Raman signal enhancement. By enabling efficient analyte excitation and enhanced scattered signal collection through waveguide-mediated light-matter interactions, this approach unlocks new possibilities for high-sensitivity Raman detection. Furthermore, we discuss the potential of this integration to drive breakthroughs in fields such as biomedical diagnostics, environmental monitoring, and chemical sensing, paving the way for next-generation, portable and ultra-sensitive sensing platforms.

## Abstract (web summary)

Surface-Enhanced Raman Scattering (SERS) integrated with optical waveguides enhances sensitivity, simplifies design for ultra-low analyte detection.

## Graphical abstract

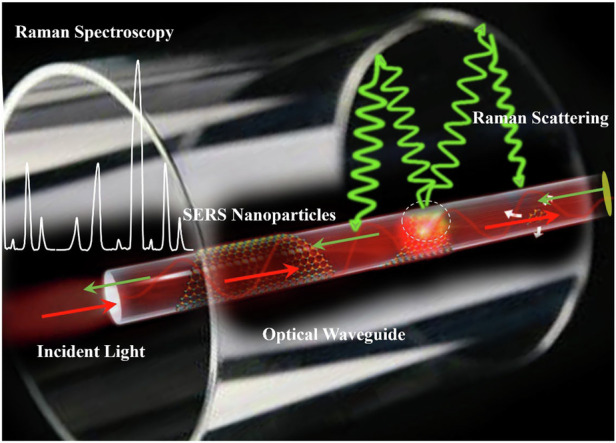

## Introduction

In recent years, the demand for highly sensitive, portable, and label-free sensing technologies has grown significantly across various fields, including biomedical diagnostics, environmental monitoring, and chemical analysis. Surface-enhanced Raman scattering (SERS) has emerged as a powerful analytical tool due to its exceptional sensitivity and ability to provide molecular fingerprint information without labeling^1–6^. SERS, discovered in the 1970s, relies primarily on the localized surface plasmon resonance (LSPR) effect^7–10^. This effect occurs at the interface of noble metal nanostructures (e.g., gold, silver, and copper) and enhances Raman signals by several orders of magnitude. These metal-based SERS substrates have been widely employed in the detection of trace analytes, offering unparalleled sensitivity in molecular identification.

However, conventional SERS techniques face several inherent limitations that hinder their widespread adoption. Traditional SERS requires coating a substrate with metal nanoparticles, depositing samples, and analyzing them using bulky Raman instruments for extended periods. This approach suffers from complex operational procedures, limited sample handling capabilities, and challenges in aligning the excitation interface with the signal collection area. Furthermore, limited spatial collection efficiency of Raman signals is often constrained, leading to suboptimal detection sensitivity, particularly for ultra-trace analytes in complex matrices^11–13^. These drawbacks, coupled with rising demand for portable and rapid sensing, have driven innovations to address these challenges.

Optical waveguides, particularly advanced optical fiber architectures, have garnered significant research interest in photonic sensing applications due to their unique combination of mechanical flexibility, compact geometry, and exceptional immunity to electromagnetic interference^14–17^. The engineering of specialized waveguide geometries enables tailored solutions for diverse detection scenarios, while strategic integration with complementary photonic technologies expands the operational versatility of optical sensing platforms^18–21^. This technological synergy has catalyzed innovative approaches in surface-enhanced Raman spectroscopy (SERS), where waveguide-mediated excitation and signal collection mechanisms overcome traditional limitations in conventional SERS configurations. Precisely engineered evanescent coupling allows waveguides to simultaneously deliver excitation and collect signals with high efficiency. This optimizes electromagnetic confinement and boosts sensitivity via spatially controlled plasmonics^22^.

Notably, microstructured waveguide platforms including photonic crystal fibers (PCFs) and lab-on-fiber devices demonstrate unprecedented analytical capabilities through monolithic integration of microfluidic functionalities^23^. These hybrid architectures enable continuous-flow, label-free analysis of attoliter-scale liquid specimens with sub-second temporal resolution, representing a paradigm shift in real-time molecular sensing applications. As Table 1 shows, waveguide-enhanced SERS outperforms conventional configurations in detection limits, multiplexing, and stability. Current technological implementations range from fiber-endface-integrated SERS probes for remote measurements to optofluidic systems with transverse microchannels that maximize analyte-waveguide interactions.

**Table 1:** Summary of advantages and disadvantages of Raman sensors based optical fiber and conventional SERS

Type	Advantage	Disadvantage
Conventional SERS detection	Enhancement of the Raman signal of the sample	In a large Raman instrument for long-term Raman detection analysis
Raman detection of the sample solution directly by the fiber optic tip	Samples can be assayed in vitro and in vivo; demonstrates flexibility of probes for easy production	Small area sensing limits progress to high detection limits
SERS substrate coated on the tip of the optical fiber for Raman detection of the sample solution	Increased detection limits for detectors	Longer scanning time required; higher laser power; large sample requirements
Collection of Raman signals from liquids or gases inside photonic crystal fibers	High detection limit; trace detection of samples	Unable to construct a microfluidic circulation feed structure for optical fiber aperture treatment

**Fig. 1:**
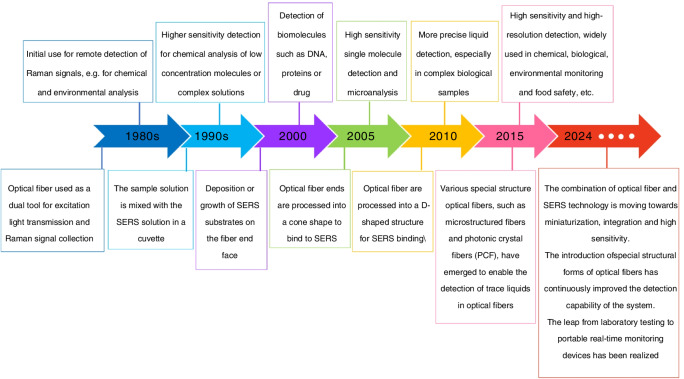
A timeline picture of the development of optical fiber sensing technology combined with Raman spectroscopy technology

This review explores the developmental history (shown in Fig. 1) and the latest advancements in combining optical waveguide architectures with SERS technology, highlighting interface optimization and substrate design for maximum Raman enhancement. We categorize these advancements into two main approaches: (1) remote sensing probes based on SERS-functionalized optical fiber tips, and (2) microfluidic SERS sensing platforms utilizing the internal structures of microstructured optical fibers. These integrated systems harness waveguide-SERS synergies to enable ultrasensitive detection, offering transformative potential for next-generation portable sensors.

Beyond summarizing existing technologies, this review identifies the limitations of current approaches and proposes future research directions. Emerging technologies, such as the integration of two-dimensional materials (e.g., graphene, MXenes) and advanced machine learning algorithms for signal processing, are discussed as promising avenues for further enhancing SERS performance. We further examine applications in optical waveguide-integrated SERS in biomedical diagnostics, environmental monitoring, and chemical industrial process control, emphasizing the need for scalable fabrication techniques and robust, reproducible sensing platforms

This review addresses key challenges in conventional SERS methods and outlines emerging technological solutions, providing researchers with a comprehensive resource for Raman sensing and optical waveguide-based detection.

### Advancements in optical waveguide-integrated SERS sensing platforms

The integration of SERS technology with optical waveguides addresses critical limitations of conventional SERS methods, such as alignment complexity, limited spatial signal collection, and bulky instrumentation. Waveguide-enabled approaches have yielded transformative approaches to SERS-functionalized fiber optic probes for remote sensing. Below, we analyze these strategies, emphasizing their design principles, performance metrics, and unresolved challenges.

### Remote SERS sensing probes: from fiber tips to advanced geometries

#### Fiber tip-modified SERS probes

**Fig. 2:**
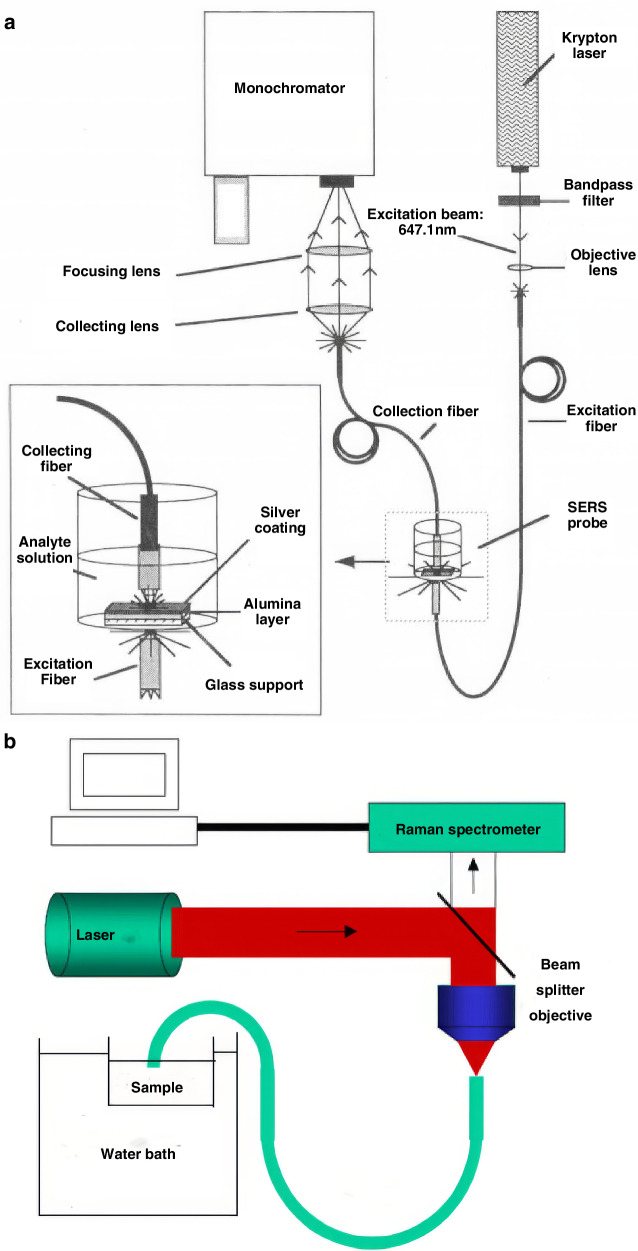
Schematic of non-contact detection of optical fiber and SERS substrate. **a** Schematic diagram of the in-situ SERS fiber optic system^24^. © ACS Publications **b** Schematic of the experimental setup^25^. © Elsevier

The earliest efforts to integrate SERS with optical fibers focused on functionalizing fiber tips with plasmonic nanostructures. In 1991, Bello et al. pioneered this approach by employing a single-mode fiber to excite and collect SERS signals from analytes mixed with colloidal nanoparticles in a cuvette, as shown in Fig. 2b^24,25^.

A typical system for non-contact excitation and reception of Raman signals between an optical fiber and a SERS substrate is shown in Fig. 2a. In this system, an aluminum layer was grown as a SERS substrate on a glass support and placed in a container containing the analysis solution. The single optical fiber strand was used to deliver the laser beam into the sampling system, and another fiber was used to collect the scattered radiation. This method uses optical fibers to excite and receive scattered light after the interaction between the SERS substrate and the sample. It can already detect liquid samples with lower concentrations, and the detection limit for (4-Aminobenzoic acid, PABA) and terephthalic acid can reach 10^−7 ^mol/L.

Although both methods enabled in situ detection, their sensitivity remained limited by poor excitation-SERS region overlap^26,27^. This spatial mismatch reduces Raman signal generation and necessitates scattered light collection through free-space optics, ultimately restricting detectable sample concentrations. Overcoming this concentration barrier remains a key research priority, as lower detection limits would significantly expand Raman spectroscopy’s applications.

**Fig. 3:**
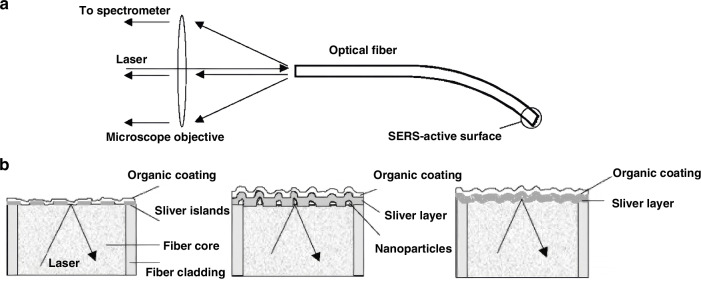
The sensing terminal is constructed by integrating the optical fiber end-face with the SERS substrate. **a** Experimental arrangement for a single-fiber SERS sensor **b** SERS substrates with different morphologies at the optical fiber end-face^31^. © Wiley Online Library

Subsequently, to improve the detection sensitivity of optical fiber-based SERS sensing, a SERS substrate was chemically grown on the tip of the optical fiber to construct a highly sensitive Raman detection probe for biological and chemical molecule detection^28–30^. Viets and Hill’s seminal work in 2000 introduced silver-coated fiber tips with tilted end faces (40° inclination), optimizing plasmonic coupling through controlled Ag/Al_2_O_3_ morphologies^31,32^. During the experiment, they also discussed the effects of the tilt angle of the fiber end face, the morphology of the SERS substrate grown on the optical fiber end-face, and the length of the sensing fiber on the Raman detection effect of the sample. Three distinct SERS architectures were compared: continuous silver films, silver nanoparticles, and aluminum-supported silver nanoparticles, as shown in Fig. 3. The 40° Ag/Al_2_O_3_ configuration yielded maximal enhancement, enabling remote detection over 95 meters.

**Fig. 4:**
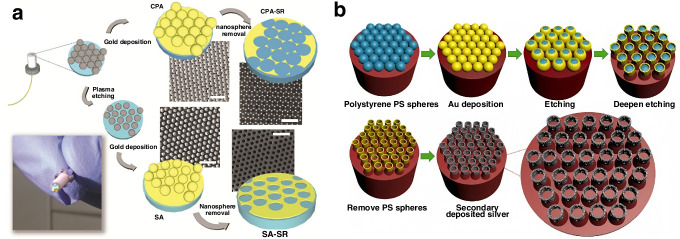
Construction of densely arranged SERS nanoparticles on the optical fiber end-face. **a** Tip of the optical fiber, coated with 30 nm gold and with the sample to be studied^33^ © 2017 SPIE **b** Schematic representing the fabrication procedure for creating different fiber tip nanoprobes and SEM images of real samples^34^ © ©Elsevier

Further innovations, such as Pisco et al.’s nanosphere lithography technique (Fig. 4a)^33^, enabled the fabrication of reproducible, large-area SERS substrates (e.g., close-packed polystyrene arrays) on fiber facets. In a common dye (crystal violet, CV) detection validation, these substrates generated dense “plasmonic hot spots,” reducing the detection limit for CV to sub-nanomolar levels.

On the other hand, the Taguenang J. M.’s group detected SERS on the tip of a 3 mm-core diameter (poly methyl methacrylate, PMMA) plastic optical fiber. The technique involves deposition of 30 nm gold nanoparticles followed by deposition of sample of interest to be analyzed^34^. The group used a focused 633 nm laser to simplify the SERS detection by connecting the other end of the optical fiber directly to the spectrometer. the interference spectrum of PMMA can be phase-subtracted to obtain the SERS spectrum of the target molecule, and finally succeeded in detecting the very low concentration of the sample solutions (rhodamine 6 G, glycerin, Streptavidin, (poly-L-lysine, PLL), concentrated acetaminophen and salicylic acid, etc.) at the end of the optical fiber, with the detection sensitivity of 0.1 pg.

Recent innovations, such as the gold nanocavity arrays decorated with silver nanoparticles by Meng et al. (Fig. 4b)^34^, further enhanced sensitivity through multi-reflective light trapping and hybrid LSPR effects. This (nanocavity ordered array, COA) structure has a strong light trapping capability and improves light utilization through multiple reflections. In addition, the COA has a large specific surface area, which can provide a large number of SERS “hot spots” and also absorb more probe molecules. In addition, the secondary deposition of silver leads to strong coupling between gold nanocavities and silver nanoparticles. Therefore, the nanocavity arrays can further enhance the localized surface plasmon resonance (LSPR) effect, thereby improving the sensitivity of the SERS electrodes. This platform achieved 0.1 pg detection limits for acetaminophen, demonstrating exceptional pharmaceutical analysis capabilities.

#### Tapered and D-shaped fiber probes

To overcome the limited evanescent field interactions in flat-tip designs, researchers have developed tapered and D-shaped fiber probes. Optical fibers with these structures enhance evanescent field exposure, increase the interaction with the sample, and obtain stronger Raman scattering signals.

**Fig. 5:**
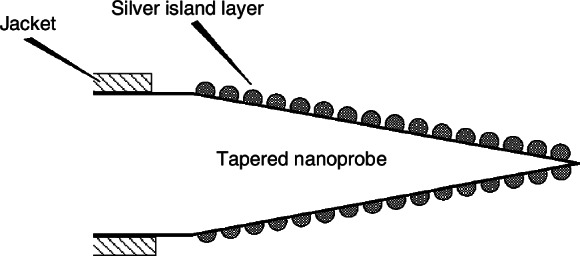
Schematic diagram of the silver islands-based SERS nano-probe35. © Sage Journal

Vo-Dinh et al. demonstrated a breakthrough with sub-10 nm tapered fiber tips coated with Ag islands (Fig. 5)^35^. In the experiment, a 20 cm long section of 600 μm-core was clamped in a 10 W CO_2_ laser based system and subjected to conical stretching. The small scale of the tip may be amenable to localized, nondestructive SERS-based analyses of surfaces with high spatial selectivity. Furthermore, an oscillating mirror defines the length of fiber heated by the laser. The pulling process involves a computer-controlled solenoid, which provides a velocity-of-pull feed-back signal to the computer. This technique offers a high degree of control over both the diameter of the tip and the length (and hence the angle) of the taper.

The contact probe’s intrinsic SERS activity enables direct, label-free analysis of surface analytes with sub-micrometer spatial resolution. This approach achieved 25% signal variation across five probe tips, demonstrating applicability to diverse surfaces without sample pretreatment.

The nanoscale tip enabled localized SERS measurements on dry surfaces, showcasing applications in in situ chemical mapping and intracellular analysis. This growth of SERS substrates at the tip of the optical fiber as Raman probes lays the foundation for the subsequent field of combining optical fiber sensing and SERS technologies^36,37^.

**Fig. 6:**
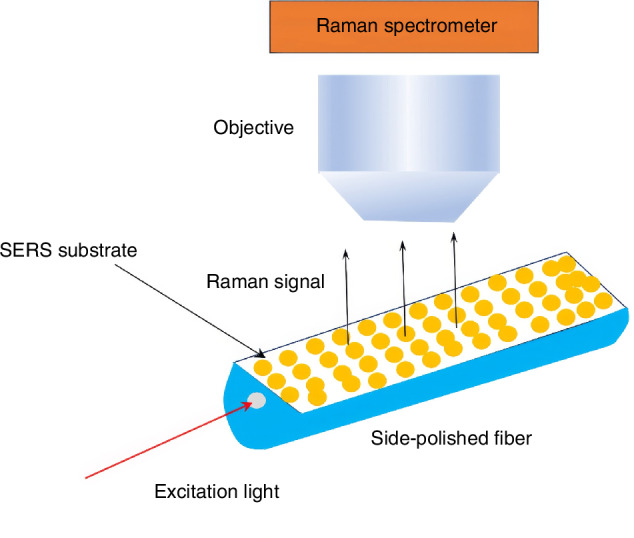
D-shaped optical fiber SERS sensor

The sparse distribution of SERS-active particles necessitates prolonged scanning with high laser power. To address this, researchers developed two complementary strategies: (1) optical fiber probes enhancing local fields, and (2) active-area expansion via D-shaped fibers (Fig. 6)^38^. Side-polished to expose the core, these D-shaped fibers provide a 1.6 × 10^3^-fold larger SERS area than conventional tips. Their unique geometry enables perpendicular excitation and intensified light-analyte interactions, yielding three-order-of-magnitude Raman signal enhancement.

### Challenge and solution

Despite their potential, current remote sensing probes face scalability challenges in medical, environmental, and chemical testing applications where trace liquid analysis demands ultrahigh sensitivity. The high consumption of liquid samples by this remote sensing probe structure, the need for precision machining, the susceptibility to mechanical damage, and the limited contact area of the optical waveguide probe with the liquid, limiting its detection sensitivity, are all challenges for scalable deployment^39^.

Optical fiber evolution has produced specialized architectures for trace analyte detection, such as photonic crystal fiber^40–42^, has gradually appeared, through its internal porous structure to realize the detection of gas in the fiber^43^; Hollow optical fiber, using the air-channel inside the optical fiber can realize highly sensitive detection of gas and liquid^44–46^; Ring core fiber, Suspended core fiber, etc. In order to meet the need for highly sensitive detection of trace liquid and gas samples, the shape of the optical fiber has gradually developed into various forms, and the optofluidic Raman sensing probe is constructed by combining with SERS to facilitate the best results^47^.

### Hollow-core PCFs for enhanced sensitivity

**Fig. 7:**
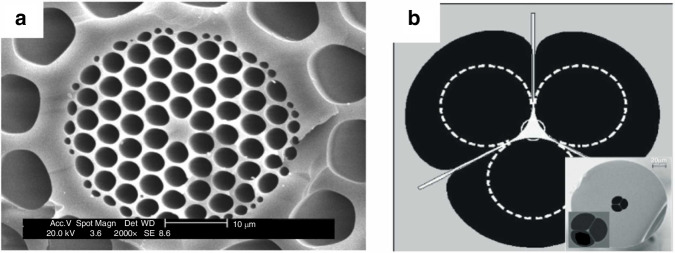
Photonic crystal optical fiber^49^. **a** Microscopic image of the cross-section of a solid silica optical fiber. **b** Scanning electron microscope (SEM) image of a suspended-core photonic crystal fiber (PCF). © Elsevier

Since their 1990s debut, photonic crystal fibers (PCFs) have emerged as a groundbreaking class of optical waveguides, characterized by their unique microstructured cladding and hollow-core designs (Fig. 7). Their hallmark ultra-low propagation loss (<0.1 dB/km in specific bandgap-guided configurations), which enables centimeter-scale light-matter interaction lengths^48^.

**Fig. 8:**
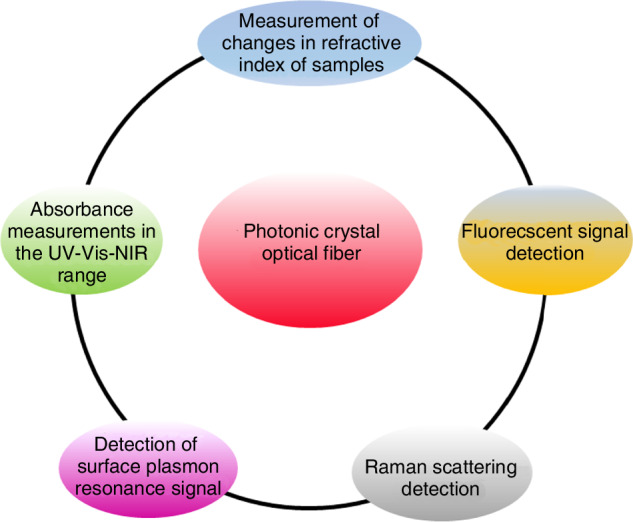
Correlation diagram of microfluidics with different analytical techniques

This extended interaction enhances analytical signals through coherent accumulation effects, particularly in evanescent-field-based sensing modalities^49^. PCFs have been extensively exploited for multiparameter sensing applications, including (i) refractive index (RI) metrology with sensitivity up to 10,000 nm/RIU^50^, (ii) broadband absorbance spectroscopy spanning UV-vis-NIR wavelengths^51,52^, (iii)surface plasmon resonance (SPR) detection at attomolar concentrations^53,54^, and (iv) fiber-enhanced Raman scattering (FERS) with signal amplification exceeding 10^6^ fold^55–57^(Fig. 8).

**Fig. 9:**
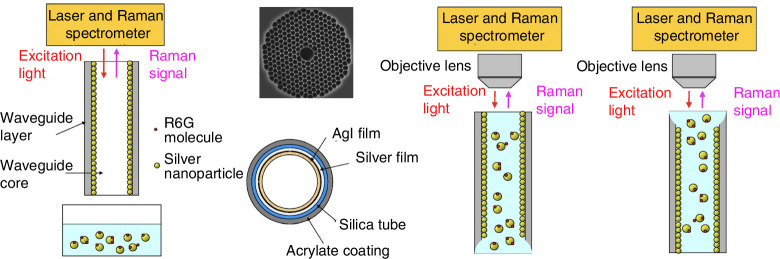
Schematic of the IWCHCW sensor^60^. © AIP Publishing

In Raman applications, PCF exploits bandgap effects to confine light in hollow cores, enabling centimeter-scale interaction paths with analytes^58,59^. Shi et al. pioneered this approach by coating hollow-core fibers with Ag nanoparticles (AgNPs), creating a “sandwich” SERS enhancement (Fig. 9)^60^. Their system demonstrated that after growing a SERS substrate on the surface of the optical fiber and placing the optical fiber probe into a cuvette with a mixture of SERS and sample solutions, the SERS nanoparticles with the samples would bind to the SERS substrate on the surface of the probe, resulting in a chemotaxis effect and increasing the Raman sensing effect of the optical fiber probe^61^. Rhodamine 6 G (R6G) was used as the analyte molecule to achieve highly sensitive detection of trace amounts of R6G at a concentration of 10^−6^ Mol/L in an effective liquid volume of 1.41$\times$10^−4^ $\mu L$. This design amplifies the R6G signal by more than 10 times compared to a planar substrate.

### Tapered opto-fluidic photonic crystal fiber with SERS

**Fig. 10:**
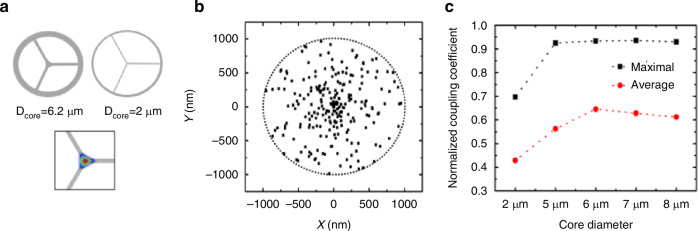
Simulation results of SuC-PCFs. **a** Design of SuC-PCFs used in the simulation with different core sizes. **b** Distribution of the center of the laser beam randomly positioned (200 times) within an area limited by a circle of 1 μm radius around the center of the fiber. **c** Maximal and average coupling coefficient values for SuC-PCFs with different core diameters^62^. © Optica Publishing Group

The Amine Benazza group developed tapered suspended-core photonic crystal fiber (Tapered-SuC-PCFs) using a stack-and-draw technique, assembling three silica capillaries in a silicone tube preform under controlled pressure gradients (Fig. 10a)^62^. Its mode field diameter (MFD) is about 2 μm, the emission wavelength is 785 nm, and the laser is linear polarization maintained through stress-induced birefringence. Based on these parameters, the overlap integrals of the SuC-PCF fundamental mode EMF distribution with the Gaussian beam of the excitation laser were calculated using the finite element method to generate a two-dimensional matrix of the components of the EMF distribution of the incident beam (linearly polarized). In order to simulate possible deviations during the measurement process, the center of the incident beam was randomly positioned at 200 points within a disk with a radius of 1 μm (Fig. 10b).

The normalized coupling coefficient (C) is derived according to Eq. (1) based on the continuity of the transverse field components between the incident beam and the fiber modes across the fiber cross-section:

1 $$C=\frac{1}{4}\times\frac{\int({\overrightarrow{E}}_{tn}(x,y)\times{\overrightarrow{H}}(x,y)+{\overrightarrow{E}}(x,y))dxdy}{\sqrt{\frac{1}{2}\int{\mathrm{Re}}({\overrightarrow{E}}_{tn}(x,y)\times{\overrightarrow{H}}_{tn}^{\ast }(x,y))dxdy}\times \sqrt{\frac{1}{2}\int{\mathrm{Re}}({\overrightarrow{E}}(x,y)\times{\overrightarrow{H}}^{\ast}(x,y))dxdy}}$$

With E_x/y_ and H_x/y_ are the x and y components of the electric and magnetic fields of the fundamental mode fluid inside the fiber. *E*_*tn*_*(x*, *y)* and *H*_*tn*_*(x*, *y)* are the *x* and *y* components of the electric and magnetic fields of the laser Gaussian beam.

Figure 10c represents the normalized coupling coefficient between the incident Gaussian beam and the fundamental mode of SuC-PCFs with different core diameters. Finite element simulations revealed a normalized coupling coefficient of 0.93 (maximal) and 0.64 (average) with the Tapered-SuC-PCF with a core diameter of 6 $\mu m$, ensuring efficient laser-to-fiber mode matching. Functionalized with AuNPs and 4-aminothiophenol (ATP), the Tapered-SuC-PCF fiber was able to detect 100 nM of ATP, a 12-fold SERS enhancement compared to conventional cuvette systems.

**Fig. 11:**
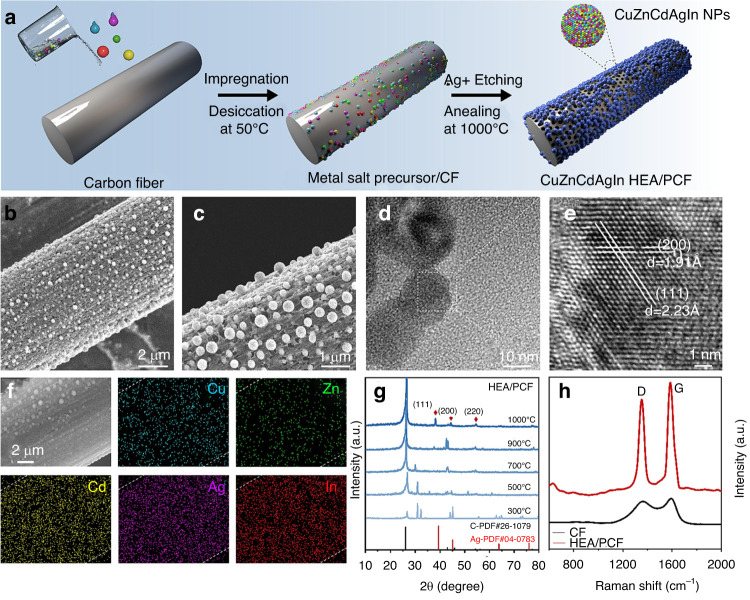
Synthesis process and characterization results of HEA/PCF. **a** Schematic illustration of the synthesis process of HEA/PCF; **b**, **c** FE-SEM and **d**, **e** HRTEM images of HEA/PCF; **f** Element mapping of HEA/PCF; **g** XRD patterns for HEA/PCF with temperatures ranging from 300 to 1000 °C; **h** Raman spectra of HEA/PCF^64^. © Elsevier

The above verifies that photonic crystal fibers (PCFs) in combination with SERS can be used as highly sensitive Raman sensing probes, therefor, broadening their applications in the detection field, mostly being used in biochemical trace sample detection^63–66^, shown in the Fig. 11.

Altaf Khetani et al. developed a portable, antibody-free leukemia detection platform using silver nanoparticle-functionalized HC-PCFs^67^. Dinish et al. created a SERS sensor based on a suspended-core photonic crystal fiber (PCF) with high reproducibility and repeatability, demonstrating the use of such a sensor for the detection of ovarian cancer biomarkers (haptoglobin) in cyst fluid, which can help to differentiate the stage of the cancer^68^_._ In 2020, this group investigated the properties of SERS and their interaction with PCF probe core size^69^. They fabricated several standard PCF design samples with different core sizes and performed SERS measurements on standard Raman-active molecules under the same conditions, allowing a direct comparison of the SERS intensity and measurement reliability for each conformation and providing a clear direction for optimizing SERS-active PCF probes.

This platform is expected to provide a unique opportunity to address several significant challenges in the development of an optofluidic liquid biopsy needle sensor capable of one-step integration of sample collection and disease diagnostic testing. However, due to the bandgap structure inside the PCFs, there are problems such as the inability to open holes to construct a fast liquid flow channel in the SERS optofluidic liquid detection probe, which can only rely on the capillary effect leading to a longer detection time; the optical mode coupling between the narrower aperture and the modes in the glass ring around the hollow core may degrade the performance^70^. These challenges highlight opportunities for developing next-generation optofluidic sensors with enhanced sensitivity and real-time monitoring capabilities.

### Hollow-core anti-resonant fibers (HcARFs): breaking sensitivity barriers

Hollow-core microstructured fibers (HC-MOF) offer an alternative to PCFs by confining light in air channels, simultaneously expanding plasmonic illumination area^71–74^ and reducing the background Raman signal from the fibrous material (glass)^75–77^. In 2022, Merdalimova et al. utilized HcARFs for nanoliter-scale SERS of R6G, resolving concentration fluctuations in drying droplets^78^. The fiber’s hollow core (240 μm diameter) confined samples within a 2 cm length, reducing reagent consumption to <5 μ*L*. This approach is scalable for liquid biopsy and exhaled air analysis, addressing clinical needs for rapid, low-volume diagnostics.

**Fig. 12:**
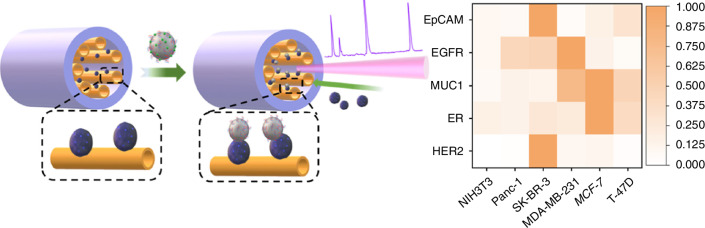
HcARF enhances the Raman scattering signal^82^. © ACS Publications

Among the various forms of microstructured optical fibers (MOF), a new hollow resonant fiber (HcARF) has recently emerged^79–81^ that utilizes anti-resonance guidance to minimize silica background signals while maximizing interaction with analytes. The light-conducting properties of this kind of fibers break through bottlenecks in the fields of high-power transmission and efficient optical waveguides and have become indispensable for biochemical, gas, strain and temperature sensing. Wang et al. demonstrated a three-order-of-magnitude Raman enhancement using HcARFs coated with Ag/ZnO nanocomposites (Fig. 12)^82^. The hybrid plasmonic-photonic coupling mechanism enabled single exosome detection, with multiplexed protein profiling on exosome surfaces. This platform achieved an enhancement factor (EF) >10^9^, critical for early cancer diagnostics.

### Suspended-core fibers (SCFs): precision sensing at the nanoscale

Microstructured optical fibers (MOFs) have advanced rapidly through innovative fabrication techniques, expanding light-matter interaction frontiers. This significant advancement in MOF is essentially due to its high degree of design flexibility, whereby fiber properties can be manipulated by simply changing the fiber geometry^83^.

**Fig. 13:**
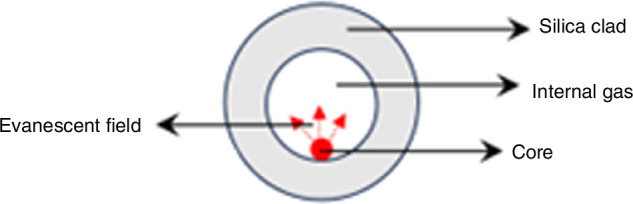
Diagram of the SCF structure^86^. © 2013 SPIE

A special example of solid core (SC) MOF is the suspended core fiber (SCF). This type of fiber consists of a silicon rod (≈1 μm) with a diameter comparable to the wavelength of light, suspended on three thin (~100 nm) struts attached to the solid cladding of the fiber. Since the pillars are much longer than the core diameter, the core is surrounded by large air holes. Due to the large refractive index contrast between the solid core and the air holes, light is confined to the core, and typically a few per cent of the total power penetrates the air holes in the form of evanescent extinction waves. Independently of the strong confinement of light, the application of optical fibers as chemical sensors is demonstrated^84,85^. L. B. Yuan’s group at Harbin Engineering University proposed a special suspended-core fiber (SCF)^86^, shown in Fig. 13, where the fiber core is suspended in the air holes near the inner surface of the capillary. It consists of three parts: a central circular hole with a diameter of ~43 μm, a suspended core of 8 μm, a ring silica cladding with a thickness of 41.5 μm, and a silicon cladding with a diameter of 125 μm. The refractive indices of the cladding, core and hole are represented by *n*_s_, *n*_c_ and *n*_h_, respectively, and *n*_c_ > *n*_s_ > *n*_d_ = 1. This type of optical fiber, leveraging its unique internal structure, holds great potential for the fabrication of evanescent field-based biosensors and gas sensors^87–92^.

Combining Raman scattering theory with optical waveguide principles, we establish the structural dependence of Raman signals in fibers. The Stokes Raman signal intensity at propagation distance z is given by:

2 $$\begin{array}{c}{P}_{D}(z)=\frac{1}{2}{n}_{E}^{hole}\frac{{C}_{j}(\lambda )k(\lambda )}{{\lambda }^{4}}{({\varepsilon }_{0}/{\mu }_{0})}^{1/2}{|{a}_{E}|}^{2}\\\qquad\qquad\qquad\qquad\times\,{\int}_{holes}{\delta }_{E}^{holes}{\mathrm{Re}}[({e}_{E}\times {h}_{E}^{{\ast}}){\hat{z}}]\exp (-{\gamma}_{E}^{z})ds\end{array}$$

3 $${\delta}_{E}^{holes}={\int}_{total}{|{e}_{E}|}^{2}ds/{\int}_{holes}({e}_{E}\times {h}_{E}^{{\ast}}){\hat{z}}ds$$

Among them, *C* is the concentration of the test sample, *λ* is the operating wavelength of the laser excitation, *j(λ)* is the Raman scattering cross-section area, *k(λ)* is the excitation beam spectral intensity, *e*_E_ and *h*_e_ are the corresponding electric and magnetic fields, *γ*_E_ is the fiber transmission loss, *ε*_0_ and *μ*_0_ are the electric and magnetic field vectors, and *Z* is the fiber length. The Raman scattering intensity is expressed by Eq. (4):

4 $$\begin{array}{l}d{R}_{R}(z)=\displaystyle\frac{\pi \exp [-\gamma {R}^{z}]}{4\omega R{\mu }_{0}{n}_{R}^{holes}{k}_{R}{N}_{R}}{\int }_{z1}^{z2}|{e}_{R}{|}^{2}{P}_{D}(z)dz\\\qquad\quad\,\,\,=\displaystyle\frac{\pi {n}_{E}^{holes}{({\varepsilon }_{0}/{\mu }_{0})}^{1/2}|{a}_{E}{|}^{2}{\delta }_{E}^{holes}}{8\omega R{\mu }_{0}{n}_{R}^{holes}{k}_{R}{N}_{R}}\frac{cj(\lambda )k(\lambda )}{{\lambda }^{4}}\\\qquad\qquad\quad\times\exp (-{\gamma }_{R}z)\exp (-{\gamma }_{E}z)dz\times {\int }_{holes}|{e}_{R}{|}^{2}{\rm{Re}}[({e}_{E}\times {h}_{E}^{{\ast}}){\hat{z}}]ds\end{array}$$

Here, *n*^holes^ is the refractive index of the liquid sample to be measured in the air hole, and *N*_R_ is the Poynting vector. Due to the small Raman frequency shift, it can be assumed that *n*_*R*_^holes^ = *n*_*E*_^holes^, *γ*_R_ = *γ*_*E*_, *k*_*R*_ = *k*_*E*_ = 2π/λ, *ω*_*R*_ = 2πc/λ, *N*_*R*_ = *N*_*E*_. Integrate the Raman signal over the entire length of the fiber, and the Raman signal strength obtained from the fiber end face is shown in Eq. 5, which can be written as the ratio of the normalized mode field area of the light field to the effective mode field area:

5 $$R=\frac{{P}_{R}}{{P}_{E}(0)}=\frac{A^{{\ast}}NOI}{{A}_{eff}}$$

The normalized overlap intensity can be expressed as where the mode field area $A$ of the optical field modes is given by Eq. 6.

6 $$A=\frac{{C}_{j}(\lambda )k(\lambda )}{8\pi {\lambda }^{2}}[1-\exp (-2\gamma )]/2\gamma$$

The normalized overlap strength *NOI* can be expressed as Eq. 7:

7 $$NOI={\left(\frac{{\varepsilon }_{0}}{{\mu }_{0}}\right)}^{1/2}\frac{{\int}_{total}{|e|}^{2}ds}{{\int}_{holes}(e\times{h}^{{\ast}}){\hat{z}}ds}\frac{{\int}_{holes}{|e|}^{2}{\mathrm{Re}}[(e\times {h}^{{\ast}}){\hat{z}}]ds}{{\int}_{total}{|{\mathrm{Re}}[(e\times{h}^{{\ast}}){\hat{z}}]|}^{2}ds}$$

The effective mode field area of light field mode${A}_{eff}$ is shown in Eq. 8:

8 $${A}_{eff}=\frac{{({\int}_{total}{\mathrm{Re}}[(e\times {h}^{{\ast}}){\hat{z}}ds])}^{2}}{{\int}_{total}{|{\mathrm{Re}}[(e\times{h}^{{\ast}}){\hat{z}}]|}^{2}ds}$$

In summary, the intensity of the Raman signal is proportional to the normalized overlap and inversely proportional to the effective mode field area. The above formula is applicable to gas and liquid Raman detection environments^93–97^. Suspended-core fiber (SCF) based sensing platforms are of particular interest for small-volume chemical and biological sensing because the signals generated by the analytes are integrated along the length of the fibers, resulting in low detection limits, while their fill volume is typically in the 10 s of nanoliters range, SCF Raman sensors have been proposed to improve Raman detection in trace liquid samples^98–101^. Meanwhile, gradually SERS techniques have been used to combine with this structure optical fiber to improve the Raman detection sensitivity^102–104^.

**Fig. 14:**
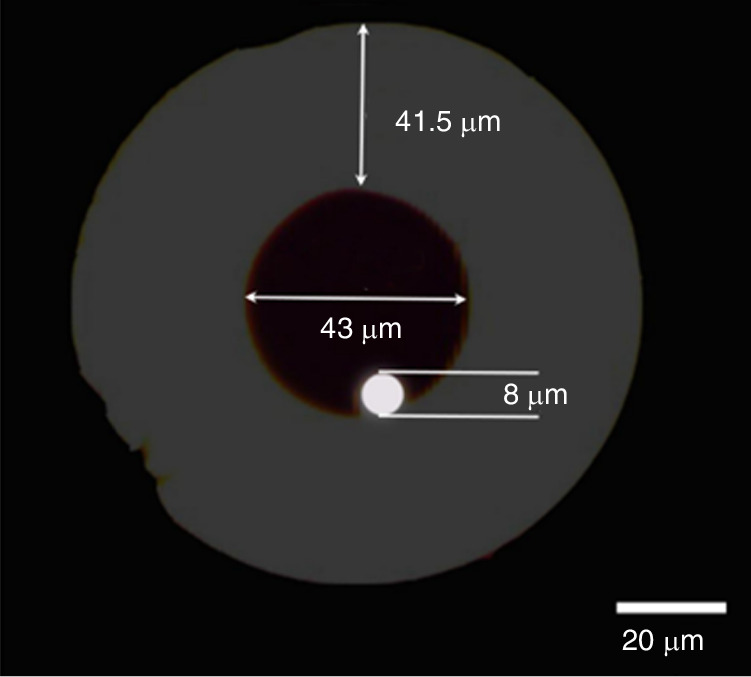
Schematic diagram of the evanescent field of the suspended core of M-SCF

In 2019, the author’s group proposed the used of microstructured suspended core optical fibers (M-SCF) in combination with SERS technology, where the fiber core is pre-treated and SERS nanoparticles are grown on the surface of the suspended core through chemical bonding connections^102^. The microscopic view of the end face of the M-SCF is shown in Fig. 14, where the sample flows inside the air holes and interacts with the SERS substrate grown on the surface of the core.

**Fig. 15:**
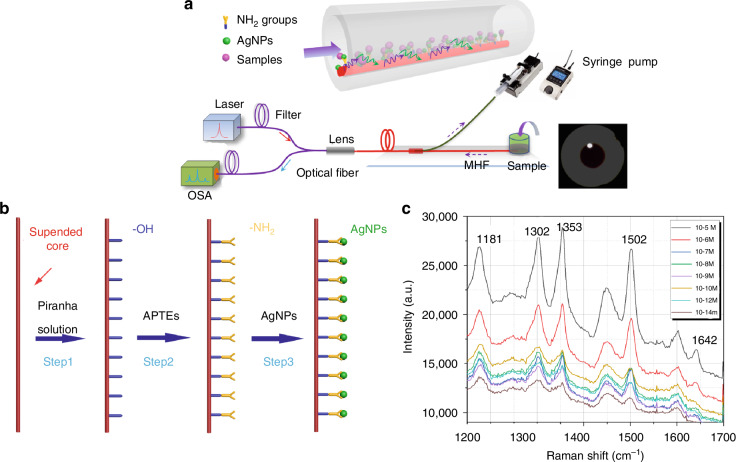
Construction of a SERS sensor using the special structure of specialty optical fibers. **a** Diagram of the device for in-fiber optofluidic SERS detection; **b** Growing process for the substrate of AgNPs on the suspended core in a M-SCF for in-fiber optofluidic SERS detection; **c** In-fiber optofluidic SERS spectra of microfluidic R6G solution (10^−14^–10^−5^ M)

Using the special internal structure of the special optical fiber, the surface of the optical fiber is treated with open holes, and the liquid sample flows through the air holes to contact with the SERS substrate on the surface of the fiber core, which excites the sample and collects the Raman signals through the evanescent field of the fiber core, as shown in the enlarged inset in Fig. 15. In the preliminary validation, using (rhodamine 6 G, R6G) as the beacon molecule, the detection limit can reach 10^−14 ^mol/L, and the calculated enhancement factor can reach 10^9^. These results imply that the Raman sensing probe constructed by combining the suspended-core optical fiber and SERS has the ability of Raman detection of trace samples with high sensitivity, which can provide a truly meaningful optofluidic Raman sensing platform for the detection of the trace chemical and biological samples in the future.

According to the comparison of the enhancement factors of several typical optical fiber structures in Raman detection, as shown in Table 2, the comparison shows that the Raman sensor with SERS substrate integrated inside this M-SCF has a high enhancement factor, which can be applied in the biological samples that are more difficult to detect.

**Table 2:** Enhancement factors of devices combining different types of optical fibers with SERS technology

Type	Detection limit for R6G	Enhanced factor
Raman detection of sample solution directly from the fiber optic tip	~10^−7 ^mol/L	10^4^–10^5^
Raman detection of sample solution with SERS substrate coated on fiber tip	10^−8^–10^−9 ^mol/L	10^5^–1.2$\times$10^6^
Collection of Raman signals from liquids or gases inside the PCFs	10^−10^–10^−11 ^mol/L	~10^8^
Raman detection of a sample solution by growing a SERS substrate inside a M-SCF	10^−14 ^mol/L	1.3$\times$10^9^

This optofluidic SERS probe based on M-SCF can achieve label-free and specific binding of trace targets through the modification of SERS nanoparticles, making it widely applicable in biological and chemical high-sensitivity detection^105–108^. Based on the above Raman probe, we designed structured SERS nanoparticles by incorporating (graphene oxide, GO) into silver nanoparticles systems, preventing aggregation while boosting Raman enhancement.

**Fig. 16:**
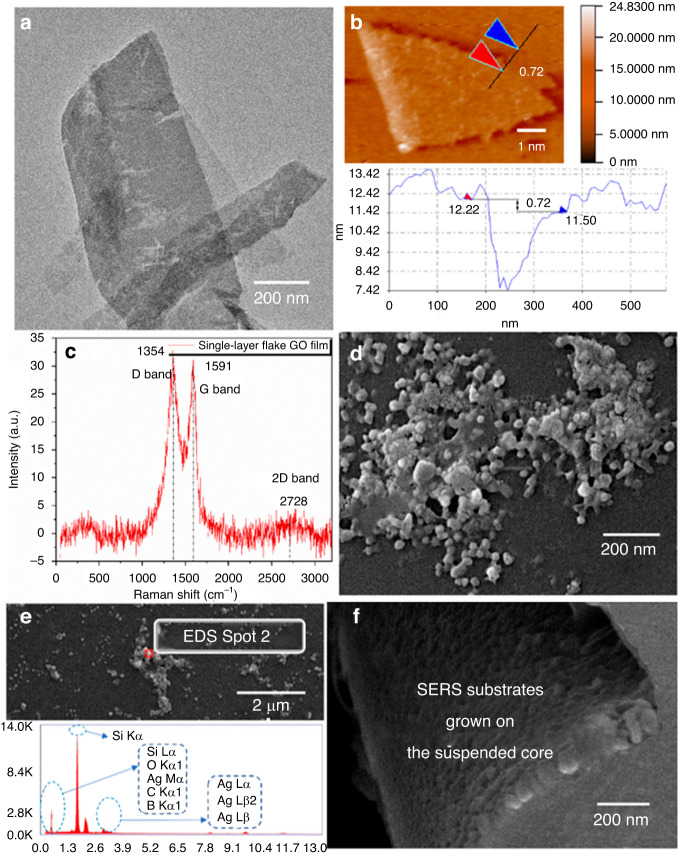
Internal characterization images of M-SCF-integrated SERS substrates. **a** SEM image of the GO solution; **b** AFM image of GO; **c** Raman spectrum of GO; **d** SEM image of the SERS substrate; **e** EDS image of the synthesized SERS substrate; **f** SEM image of the GO/Ag NPs/4-MPBA substrate modified on the suspended core^105^. © ACS Publications

Functionalizing the SERS substrate with 4-mercaptophenylboronic acid, the connection of ester bond with glucose molecules was formed, and the highly sensitive detection of glucose was achieved in the cerebrospinal fluid of SD rats to exclude the interference of other impurities. In the experiment, the concentration of glucose solutions selected was detected in the range of 0.5–10 mmol/L. In addition, the detection time was 25 s for all the different concentrations of glucose solutions. Figure 16 demonstrates the characterization of composite SERS substrate electron microscopy, atomic force microscopy, etc^105^.

**Fig. 17:**
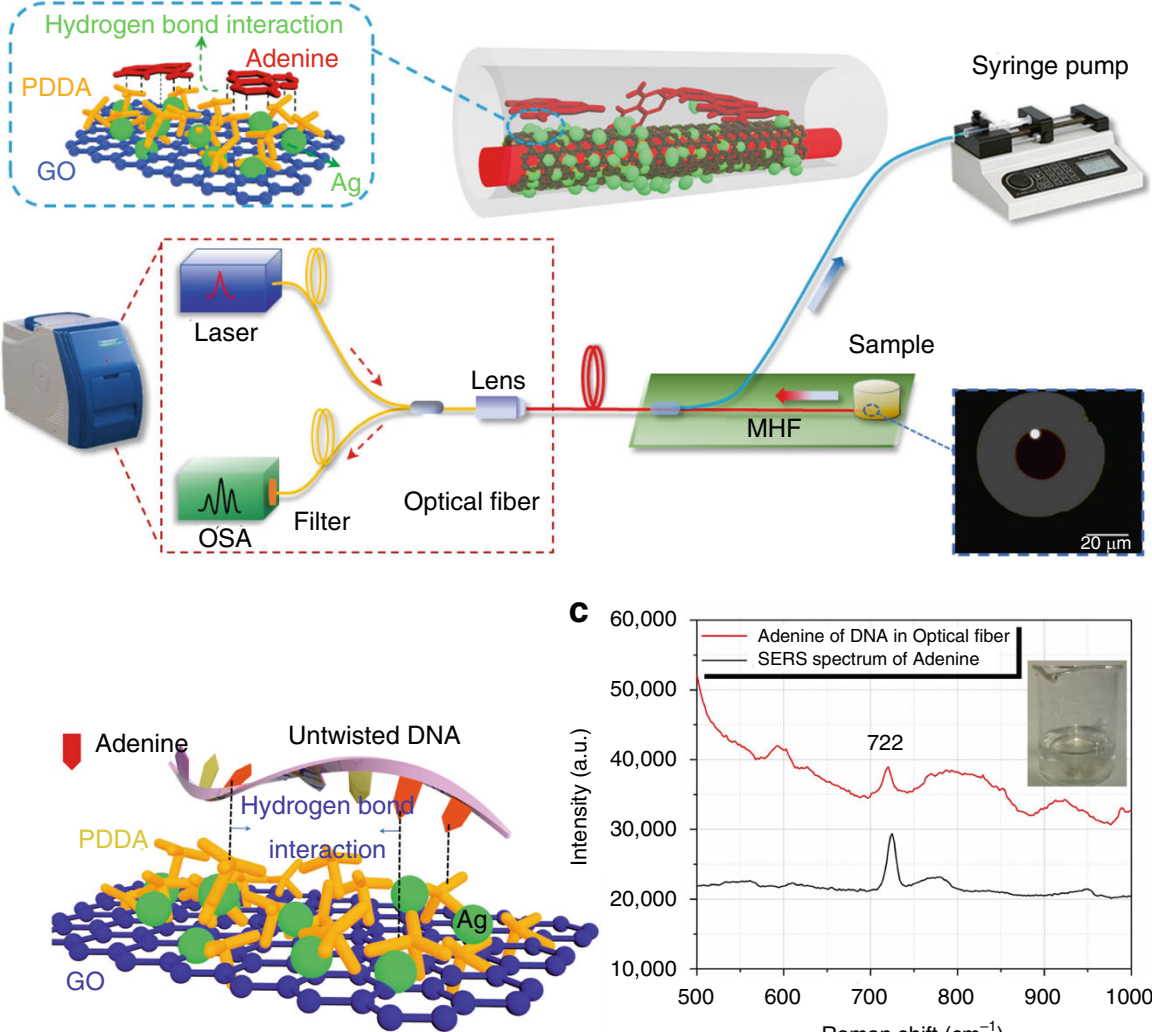
Adenine SERS detection device based on M-SCF. **a** The diagram of the in-fiber optofluidic SERS detection device. Inset: the end face of the waveguide core in M-SCF; **b** Schematic diagram of the reaction between DNA and GO/PDDA/Ag SERS substrate; **c** The Raman shift spectrum of adenine of DNA in the micro optofluidic in-fiber^106^. © Elsevier

As a fundamental DNA nucleobase, adenine is involved in the storage of genetic information and signaling in cells. If abnormal changes in the concentration of DNA bases occur, it may indicate the presence of HIV infection, hematologic disorders, or cancer in the body. Therefore, in this sense, using nano-biosensing technology to detect adenine faster and more efficiently is of great significance in clinical diagnosis. The author’s group added PDDA to the Ag NPs/ (graphene oxide, GO) solution and grew a composite SERS substrate inside the M-SCF^106^. By forming an ester bond with adenine molecules, the group achieved precise capture of adenine in crude DNA purification solutions while excluding interference from other impurities. In the experiments, the detection limit for adenine aqueous solutions reached 10^−14 ^mol/L, with a detection time of only 30 s, as shown in Fig. 17.

Bilirubin is normally excreted by the body in the form of bile; prior to this, bilirubin molecules bind to albumin to form a water-soluble complex that is present in the blood. Free bilirubin in human serum is not normally secreted and is toxic to the body. High concentrations of free bilirubin can cause a range of disorders, particularly jaundice, which is one of the leading causes of neonatal mortality. The free bilirubin level in the blood of a healthy person is less than 25 μmol/L. However, when a person suffers from jaundice, the free bilirubin level can be higher than 50 μmol/L. Therefore, the monitoring of free bilirubin concentration in the serum of a person is urgently needed for the diagnosis of a series of diseases such as jaundice. In recent years, SERS has received increasing attention due to its high sensitivity, simplicity and flexibility. In this study, this rapid, sensitive and unlabeled in-fiber free bilirubin in blood serum on-line SERS sensor can successfully detect the content of bilirubin in the actual blood environment of the human body, which provides a broad scope for accurate clinical diagnosis of jaundice and related diseases prospects. Also, due to these advantages, it has been applied in the improvement of biomedical (point-of-care testing, POCT) techniques. In the author’s group, a self-assembled GO/Ag NPs SERS sensor based on a novel photo-fluidic MHF is proposed as a POCT device.

**Fig. 18:**
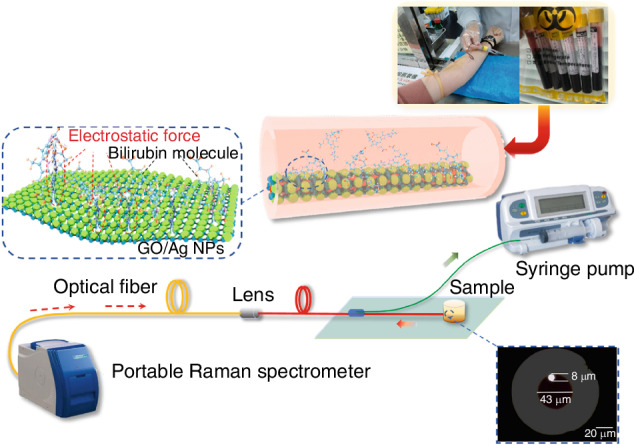
The diagram of the in-fiber optofluidic SERS detection device. Inset: the end face of the waveguide core in M-SCF^107^. © Elsevier

On the other hand, there exists electrostatic force in this kind of composite SERS (GO/PDDA/Ag NPs) substrate, and in the research, it is found that this electrostatic force can bind with bilirubin molecules and make them detached from hemoglobin. In the experiments, the bilirubin concentration ranged from 10-100 μmol/L, and the response time was bit 25 s. So that is realizes highly sensitive and rapid bilirubin detection in blood environment, and provides a research basis for the detection of diseases such as jaundice, detection of jaundice and other diseases to provide a research basis, as shown in Fig. 18^107^.

**Fig. 19:**
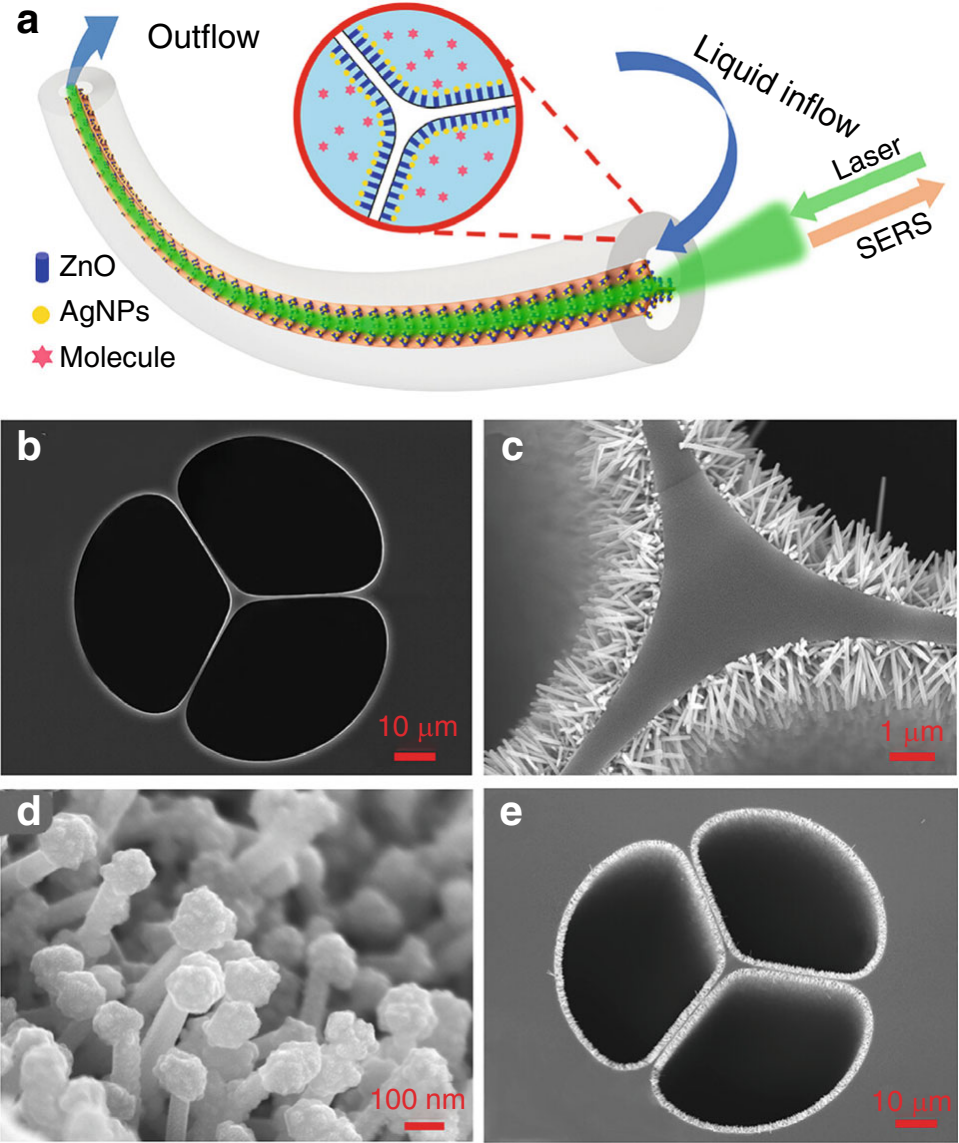
Novel sensing device with Ag/ZnO nanocomposites constructed inside M-SCF. **a** Schematic illustration of the SERS probe based on Ag/Zn Onanocomposite (AZO)-decorated M-SCF. The Raman signal was collected in a backscattering configuration. **b** SEM image of the end face of pristine M-SCF. **c** SEM image of the end face of SCF decorated with ZnO nanorods. **d**, **e** SEM images of the end face of M-SCF decorated with AZO at different magnifications^108^. © ACS Publications

Researchers are increasingly developing specialty optical fibers integrated with composite SERS nanoparticles to enable label-free, rapid, and ultrasensitive detection. In 2023, Zhang’s group proposed that the SERS platform based on a M-SCF decorated with Ag/ZnO nanocomposites on its inner surface for direct, ultrasensitive and reusable analyte detection^108^. This unique configuration not only delivers the core-located field to the liquid interface, which greatly enhances the light-analyte interaction, but also facilitates charge transfer, further improving the SERS detection sensitivity and degradation efficiency. The detection limit of the crystal violet solution was 10^−13 ^mol/L with an enhancement factor of 10^11^, as shown in Fig. 19. The relative standard deviation (RSD) was as low as 5.4%, which ensured the reproducibility of the SERS assay. The probe exhibits dual functionality, combining photocatalytic molecule degradation (20 min under UV irradiation) with ultrasensitive SERS detection. This ultra-sensitive, reusable SERS probe has great potential for rapid in situ liquid detection.

### Introduction of emerging technologies to promote practicality

In the research field of optical waveguide-SERS integration, it is essential to design specialized optical waveguide structures integrated with novel SERS nanoparticles. This approach aims to develop probes with higher Raman enhancement effect, thereby expanding their applications in trace detection, high-sensitivity, and rapid monitoring. The ultimate goal is to advance optofluidic SERS waveguide sensing technology toward practical implementation. Current challenges include optimizing the fabrication processes of SERS-active nanostructured substrates, improving the efficiency of scattered signal collection, and enhancing spectral processing precision for complex data. By incorporating cutting-edge technologies, these improvements could yield probes with superior sensitivity for Raman enhancement, transforming them into fast, efficient, in-situ detection tools for clinical diagnostics, environmental monitoring, and related fields.

### Femtosecond laser processing technology: enabling customized preparation of SERS substrates

Due to the small size of the MOF, the preparation process is more difficult for deposition as a method, and most of the researchers choose to chemically pre-treat the fibers and in-situ grow the SERS substrate inside the MOF by means of a chemical bonding connection; however, in order to ensure the homogeneity of the SERS integration in the MOF, the After a large number of experimental conclusions the growth time is strictly controlled to ensure the uniformity of the grown SERS.

Structural uniformity constitutes a paramount criterion in the systematic evaluation of surface-enhanced Raman scattering (SERS) substrates, particularly when deployed for quantitative analyte detection and hyperspectral imaging applications. The reliability of spectroscopic readouts fundamentally depends on the signal reproducibility inherent to substrate architectures. This dependence arises from the intrinsically surface-sensitive nature of SERS phenomena, which exhibit acute reliance on both localized electromagnetic field enhancement and chemical environment characteristics. For colloidal nanoparticle-derived substrates, achieving morphological homogeneity in nanoparticle dimensions, geometric configurations, and interparticle spacing is critical for ensuring electromagnetic hotspot uniformity. For engineered assembly-based substrates and periodic plasmonic arrays, precise control over interparticle gap dimensions (<5 nm) and their statistical distribution is required to optimize hotspot density and electromagnetic coupling efficiency. State-of-the-art nanofabrication methodologies, including photolithographic patterning^109,110^, electron-beam lithography (EBL)^111–115^, and focused ion beam (FIB)^116–120^ milling, have been rigorously explored to address these nanoarchitectural requirements. Nevertheless, challenges persist in simultaneously optimizing spatial uniformity, fabrication throughput, nanoscale precision, process reproducibility, and technical complexity within scalable manufacturing paradigms.

**Fig. 20:**
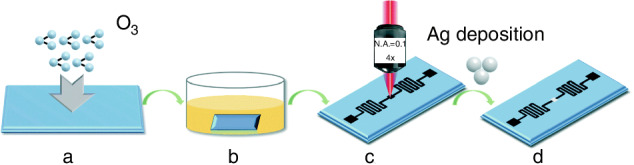
Schematic for the fabrication of the Si microfluidic SERS chips^126^. **a** Ultraviolet-ozone cleaning system. **b** Silicon wafers immersed in n-hexane. **c** Femtosecond laser fabrication of micro-nano structured square regions. **d** Silver (Ag) film deposition on square regions. © RSC Publishing

Femtosecond laser direct writing is one of the surface patterning techniques employed as a promising approach for creating three-dimensional nanostructures^121–123^. The resolution of femtosecond laser-induced forward transfer formed plasmonic particles is as high as 40 nm and the fabrication scale can reach several millimeters, enough to meet large-scale SERS detection applications, showing great potential to be a substitute for other-technique built-in Raman active substrates for microfluidic SERS application after transferring into soft polymers^124,125^. Guo’s group presented a novel self-driven microfluidic surface-enhanced Raman scattering (SERS) device for rapid detection of Hg²⁺. The device is fabricated by femtosecond laser direct writing, and does not rely on external driving sources (as shown in Fig. 20). Instead, it uses capillary action to drive the flow of the sample, with the flow speed being tunable. The SERS active detection sites are created by femtosecond laser-induced periodic surface structures (LIPSS), followed by the deposition of a 30 nm silver layer to enhance the Raman signal. The chip is used for the quantitative analysis of Hg²⁺, achieving a detection limit as low as 10^−9^ M^126^.

**Fig. 21:**
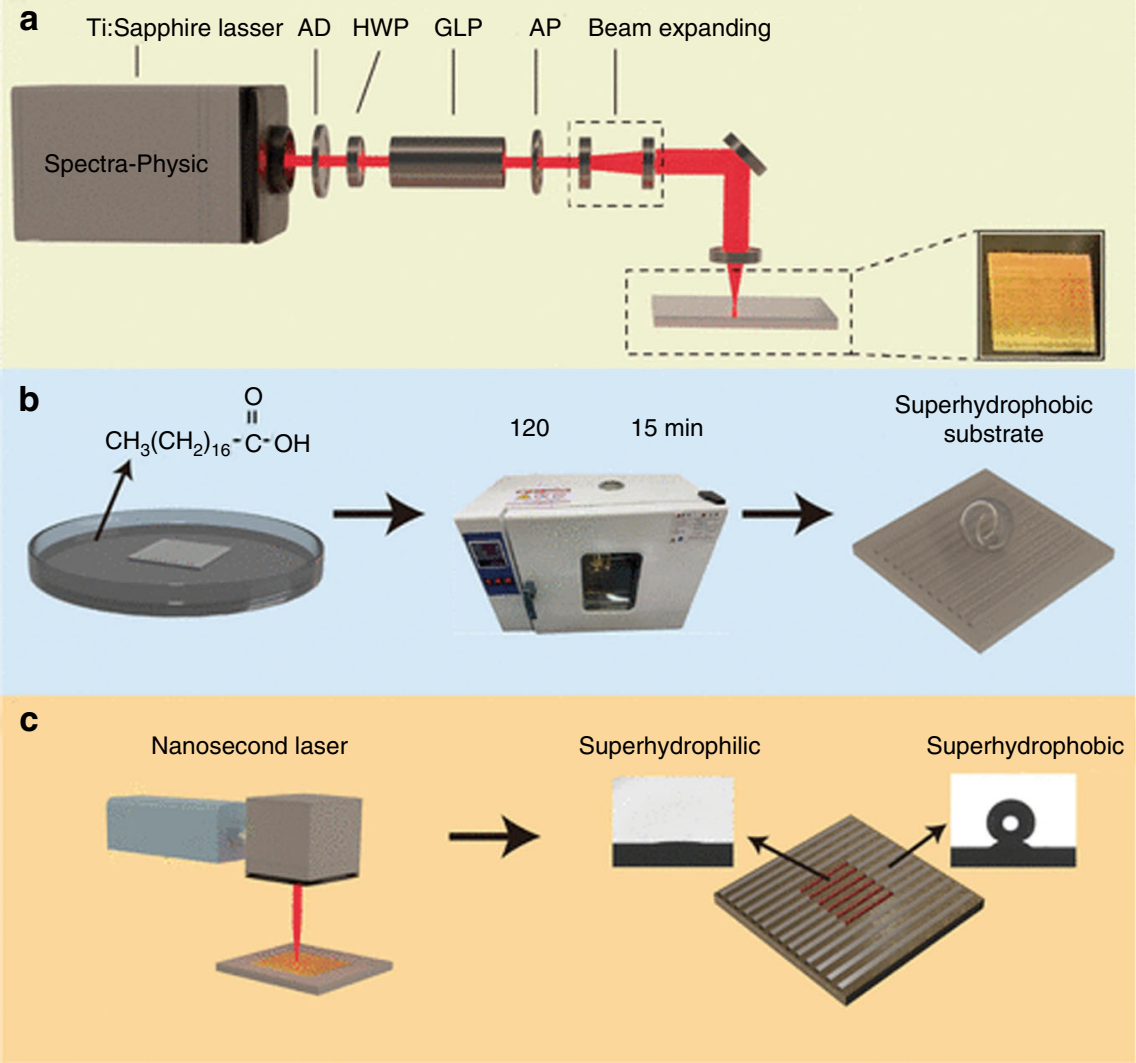
Femtosecond laser-induced SERS platform for trace molecule detection. Schematics of **a** the femtosecond laser processing setup to fabricate periodic surface structures, **b** steps to prepare a superhydrophobic stainless steel substrate, and **c** the creation of a hybrid superhydrophobic/-philic platform^127^. © ACS Publications

Hu’s group introduced a superhydrophobic/-philic surface-enhanced Raman scattering (SERS) platform based on femtosecond laser-induced periodic surface structures (LIPSS) and silver nanoparticles (AgNPs) for trace detection of molecules^127^, as shown in Fig. 21. The platform was used for trace detection of crystal violet mixed with silver nanoparticles, achieving a limit of detection (LOD) of 1.22 × 10^−15^ M and an enhancement factor of 3.69 × 10^10^. Furthermore, by integrating COF@Ag composites, the detection efficiency was significantly improved, enabling the successful detection of the antibiotic amoxicillin (AMX) with an LOD of 1.01 × 10^−11^ M. This study demonstrates the practical application potential of the superhydrophobic/-philic SERS platform in biosensing and quantitative analysis.

**Fig. 22:**
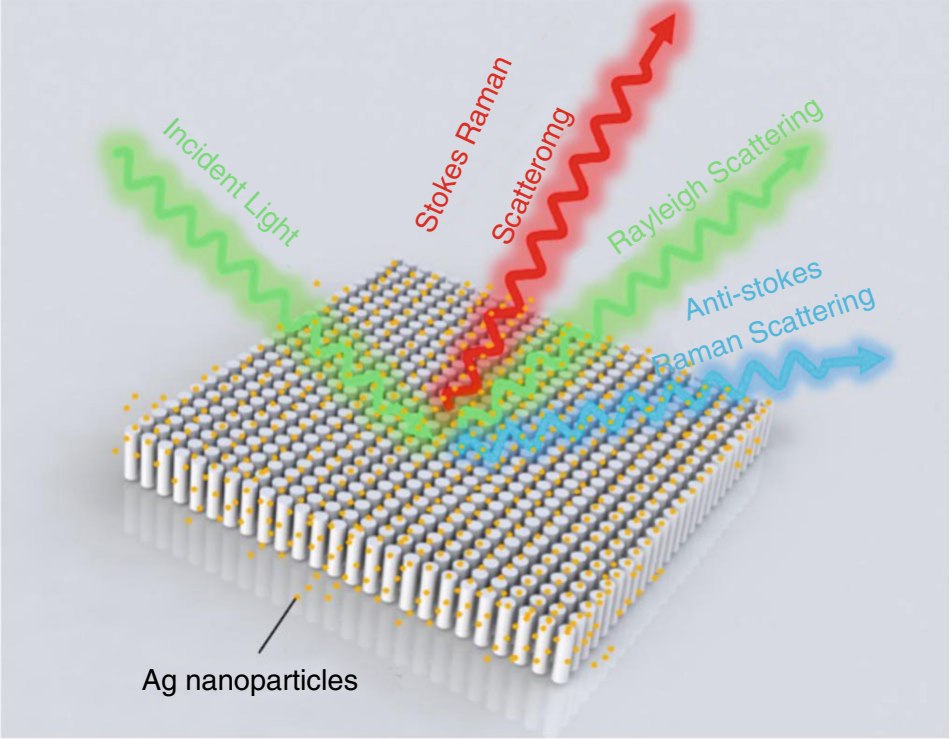
Schematic diagram of Raman scattering enhancement generated by nanopillar arrays^131^. © Elsevier

Researchers have used femtosecond laser technology to fabricate nanostructures with periodic patterns on different material surfaces, thereby enhancing the SERS signal enhancement factor and achieving high-sensitivity molecular detection, shown in Fig. 22. Sugioka’s group induced nanostripes and nanopillar arrays on ZnO surfaces using femtosecond laser^128^, combined with defect regulation and silver nanoparticle deposition, achieving a SERS enhancement factor of 2.28 × 10⁷. Venkatakrishnan’s group pioneered femtosecond laser-induced isotope enrichment at the nanoscale (LIIEN), combined with the plasma centrifugal effect, to achieve a 5000-fold enrichment of ^13^C in carbon nanostructures, providing a new method for isotope-labeled SERS detection^129^. Zhou’s group precisely controlled the femtosecond laser-induced plasma-assisted ablation (LIPAA) to fabricate silicon nanoparticles, subsequent silver coating formed a homogeneous SERS substrate with a detection limit of 1 ppm for malachite green, outperforming traditional chemical synthesis^130^. Further optimization of these substrates will expand SERS applications more widespread in the medical, environmental, and food fields, offering higher detection accuracy and real-time capabilities^131–134^.

### Cavity enhancement technology: enhancing light-matter interactions

The interaction area between light and matter determines the enhancement of sample excitation signals by integrated SERS sensing structures. To increase the interaction area between light and matter, the developmental trajectory of combining optical waveguides with SERS technology has been summarized, culminating in the construction of optofluidic sensing probes with SERS substrates integrated within specially structured optical waveguides. By leveraging the synergistic relationship between photonics and microfluidics in optofluidic technology, the interaction between light and the SERS substrate is enhanced, thereby further improving detection sensitivity and stability. However, at the current stage, the primary challenge in the research of optofluidic SERS probes revolves around enhancing the utilization of the optical field. Specifically, it is crucial to develop approaches that simultaneously increase light-matter interaction while improving SERS signal collection efficiency. To overcome traditional limitations, advancing beyond one-dimensional signal collection to achieve multi-angle sample excitation and efficient multi-perspective signal detection is essential.

**Fig. 23:**
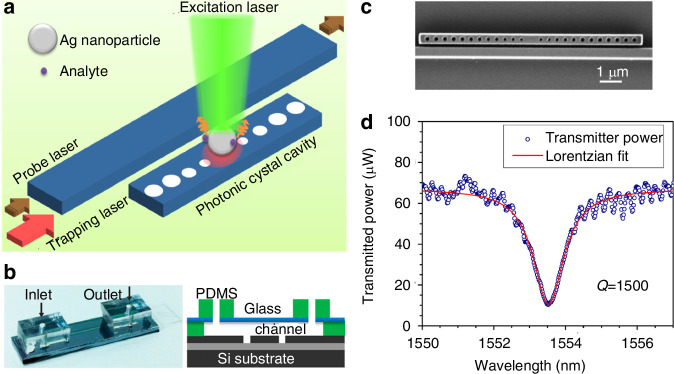
Trapping-assisted SERS platform. **a** Schematic diagram of trapping-assisted SERS platform. **b** Left: photograph of fabricated device that integrates microfluidic channel with silicon photonic chip. Right: schematic diagram of device cross section. **c** SEM image of silicon photonic crystal cavity coupled to waveguide with a gap of 200 nm. **d** Measured transmission spectrum and Lorentzian curve fit, showing a Q of 1500^139^. © ACS Publications

Notably, the integration of optical cavities with Raman spectroscopy demonstrates advantageous characteristics in optimizing the collection efficiency of Raman scattering^135–138^. On one hand, optical microcavities can enhance the local electric field energy through multiple reflections, enhancing electromagnetic hot spots on the substrate. On the other hand, they improve the optical matching between the probe and the scattered light field, enabling highly sensitive detection of target analytes. Crozier et al. fabricated a high-Q (Q ≈ 1500) photonic crystal cavity and waveguide on an SOI wafer, integrated with a PDMS microfluidic channel to form an on-chip sensing platform, as illustrated in Fig. 23. By leveraging the strong evanescent field of the TM mode to enhance optical forces, they achieved directional trapping and aggregation of silver nanoparticles. Combining the dual enhancement effects of localized surface plasmon resonance (LSPR) and cavity resonance, an average enhancement factor (EF) of 5 × 10^5^ was obtained. The platform addressed the issue of non-reusability in traditional SERS substrates by toggling the trapping laser to release nanoparticles and subsequently capture new ones, enabling sequential detection of multiple-analytes (e.g., pMA and 2-NT)^139^.

**Fig. 24:**
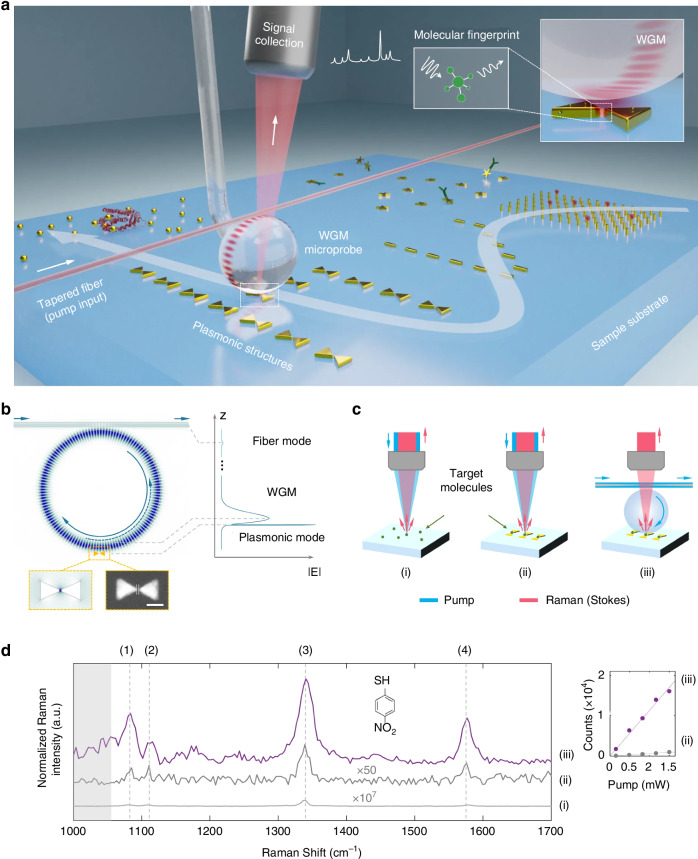
The microsphere WGM resonator is mounted on a 3D nano-translation stage through a supporting fiber stem for scanning a sample substrate^154^. **a** Schematic of a scanning whispering gallery mode (WGM) microprobe for ultrahigh-sensitivity molecular detection and imaging. **b** Numerical simulation diagram of the optoplasmonic hybrid mode. **c** Different configurations for exciting Raman scattering. **d** Raman spectra corresponding to the three configurations in c. © Springer Nature

As a prominent example, ultra-high quality factor (Q) whispering-gallery-mode (WGM) microresonators^140^, as a type of optical microcavity, are ideal candidates for sensors^141,142^, microlasers^143,144^, and nonlinear optical systems^145^. In WGM microsensors, the long photon lifetime and strong spatial confinement of light, characterized by high Q factors and small mode volumes V, respectively, enhance the interaction between light and analytes, thereby achieving high sensitivity. To further improve the detection limit, plasmonic nanostructures, such as gold nanorods, have been introduced to enhance the light-matter interaction on the surface of WGM microsensors. Studies have demonstrated single-molecule/atomic ion detection in WGM-nanoplasmonic hybrid systems^146–148^. The application of microcavities (WGMs) and optofluidic technology in Raman detection can significantly enhance the sensitivity of Raman signals, enabling applications in protein detection^149–151^ and trace volume analysis^152,153^ (e.g., pesticide residue detection). As shown in Fig. 24, Yang et al. proposed a novel scanning WGM microprobe that addresses the challenge of simultaneously achieving ultra-high sensitivity and large detection areas in traditional microsensors by leveraging the synergistic enhancement mechanism of high-Q WGM microcavities and nanoplasmonics. Through the coupling of the localized resonant optical field of WGMs and the electromagnetic field enhancement of nanoplasmonics, the sensitivity of Raman signals was improved by two orders of magnitude compared to traditional surface-enhanced Raman spectroscopy (SERS) substrates (with a total enhancement factor of 10⁸), while reducing the optical power requirements. This approach is suitable for ultrasensitive detection of proteins (e.g., BSA, CRP) and small molecules (e.g., pNTP). By fabricating periodic nanostructures using semiconductor processes, the system can be customized to enhance signals in specific wavelength bands, providing a new platform for nonlinear optics and quantum sensing^154^.

**Fig. 25:**
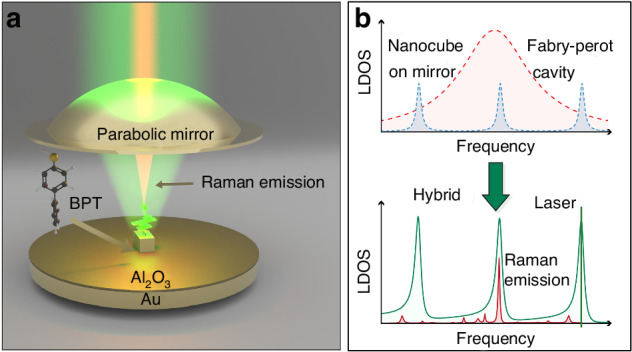
The nanocube in an FP hybrid resonator^158^. © American Association for the Advancement of Science

Beyond WGM systems, researchers have explored alternative optical microcavities for Raman enhancement. One approach involves designing SERS substrates to induce cavity-like oscillations between metal nanoparticles, generating enhanced “hot spots” and thereby amplifying the Raman signal^155,156^. Another approach involves constructing Fabry-Pérot (FP) cavity structures, which offer unique advantages in light-matter interactions. FP resonances can create wavelength-matching structures for both excitation and scattered light, offering significant potential for shaping optomechanical interactions between light and molecular vibrations^157,158^. Koenderink et al. proposed a novel hybrid cavity-antenna architecture by coupling a metal nanocube-on-mirror (NCoM) plasmonic antenna with a tunable Fabry-Pérot (FP) microcavity, achieving highly selective sideband-enhanced molecular Raman scattering^159^. The plasmonic antenna (NCoM) provides ultra-strong electromagnetic field localization through a nanogap (~6 nm) with a local density of states (LDOS) reaching 2.4 × 10^4^, while the FP microcavity (Q ≈ 300) offers spectral selectivity through its high-Q, narrow linewidth (≈1 nm). Modal field coupling creates a hybrid mode synergizing plasmonic enhancement with cavity spectral purity (mode volume 10^−4^λ^3^). In the experimental design, by tuning the FP cavity length (free spectral range FSR ≈ 110 nm) to match molecular vibrational frequencies (e.g., 1079, 1281, and 1586 cm^−1^ of BPT), selective enhancement of specific Raman spectral lines was achieved (shown in Fig. 25). The team, by designing an optimized cavity structure and integrating it with the SERS substrate, established a cooperative enhancement mechanism, ultimately achieving an enhancement factor of up to 10¹⁰, effectively suppressing background noise while resolving vibrational spectra with precision-establishing a versatile molecular optomechanics platform.

Cavity-enhanced technology offers the potential to improve the detection sensitivity of optofluidic waveguide SERS probes. In 2021, Zhang et al. proposed a Raman sensor based on a cavity-enhanced, Ag-nanoparticle-decorated tapered fiber. The Raman enhancement is primarily attributed to the localized surface plasmon resonance (LSPR) effect of the AgNPs decorated on the surface of the tapered fiber and the additional reflection of laser excitation induced by the capillary-based reflective cavity. The enhancement factor can reach up to 5.51 × 10^4^, establishing a robust framework to quantify the full coupling efficiency of the tapered fiber SERS probe and the cavity enhancement factor of the cavity-enhanced Raman sensor^160^.

Extending this team’s foundational work, the integration of cavity structures with full-length optofluidic SERS probes becomes feasible. Researchers can design appropriate cavity structures based on the wavelengths of the excitation light and scattered photons. The synergistic combination of cavity enhancement and optofluidic technology enables efficient sample excitation and multidimensional scattered-photon collection, significantly boosting Raman enhancement effects. This significantly enhances the Raman enhancement effect, enabling the development of cavity-based optofluidic Raman sensing probes with higher detection limits. The approach not only facilitates high-sensitivity detection at the single-molecule level but also paves the way for practical applications.

### Machine learning: assisting manual analysis of spectra

In Raman spectral analysis, the complexity of Raman spectral features of biological macromolecules exhibiting multiplexed spectral signatures in complex matrices and the complexity of the composition of biological samples pose a great challenge to accurately extract meaningful information. Multivariate analysis and machine learning (ML) methods have been employed to analyze these spectral datasets^161^.

**Fig. 26:**
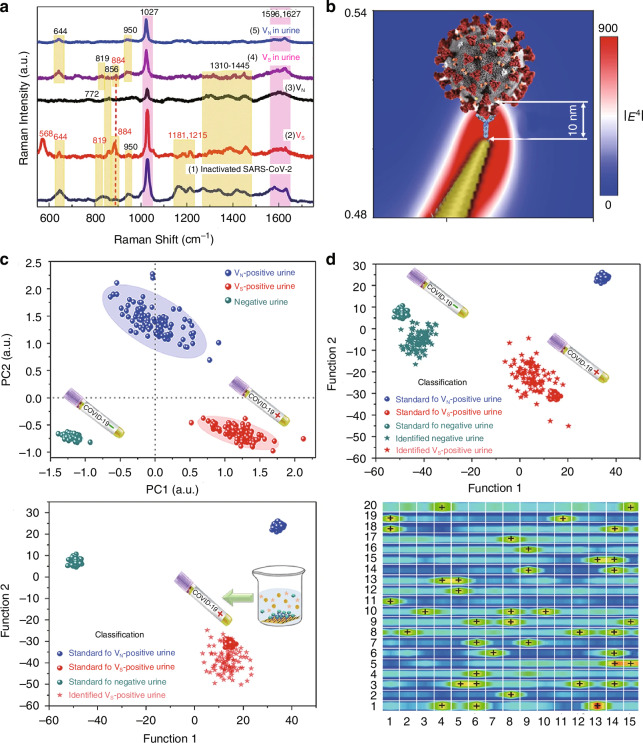
Establishing and validating the SARS‑CoV‑2 virus identification standard based on machine‑learning method^167^. **a** Surface-enhanced Raman spectroscopy (SERS) spectra of inactivated SARS-CoV-2, virus VS, and virus VN (with a viral load of 2200 copies/mL) in phosphate-buffered saline (PBS) and urine from an 8-year-old healthy girl. **b** Schematic diagram showing the localization of the SARS-CoV-2 spike protein (S protein) within the 10 nm electromagnetic (EM) enhancement region, and the calculated electric field intensity distribution (|E|²) at a wavelength of 785 nm at the tip of an oblique gold nanoneedle. **c** Key features of SERS patterns for distinguishing VS virus-infected urine samples (simulated water samples contaminated with SARS-CoV-2 virus), VN virus-infected urine samples, and urine samples from healthy individuals via principal component analysis (PCA). **d** Discriminant analysis (DA) results for identifying urine samples from patients with chronic nephritis and urine samples from patients with chronic nephritis containing virus VS. The green, red, and blue spheres represent the negative urine standard, VS virus-positive urine standard, and VN virus-positive urine standard, respectively. Discriminant analysis (DA) results for identifying virus VS and virus VN mixed in adult urine (with a viral load of 2200 copies mL^−1^). SERS mapping image (40 × 30 μm²) of a urine sample covering 300 measurement points, among which 42 dot-measurement areas can be identified as VS virus-positive. © Springer

Deep learning methods using complex artificial neuron structures enable advanced feature and pattern recognition. Among them, Convolutional Neural Networks (CNNs) show higher specificity and sensitivity by using shared weight filters and pooling layers in their architecture^162,163^. When evaluating a model, where the sample size is not sufficient to form a large test set, cross-validation can be used to evaluate model performance by omitting a validation set during training and constructing multiple permutations of the training and validation sets, e.g., K-fold multiple cross-validation, leave-one-out method, etc^164–166^. The use of cross-validation must be handled carefully by selecting representative features (variables) or adding a large sample size based on the complexity of the features otherwise, it will be easy to generate overfitted models, i.e., high performance on the training set but poor performance on the test or validation set. Luo’s group developed a surface-enhanced Raman scattering (SERS) biosensor based on human ACE2-functionalized gold nanostructures for highly sensitive and rapid detection of SARS-CoV-2. By modifying ACE2 receptors onto obliquely aligned gold nanoneedle arrays (GNAs), the sensor utilizes the specific high-affinity binding between ACE2 and the viral spike (S) protein (10–20 times stronger than SARS-CoV), in conjunction with the “virus-trap” nanoforest structure of GNAs and plasmonic effects (enhancement factor up to 10⁹), achieving single-molecule-level viral capture and detection. Additionally, machine learning algorithms (Principal Component Analysis, PCA, and Discriminant Analysis, DA) were employed to further optimize the virus signal recognition criteria, establishing a scalable detection methodology. This approach provides a paradigm for the rapid detection of future unknown coronaviruses, as illustrated in Fig. 26.

Raman spectral analysis requires systematic preprocessing involving baseline correction, spectral smoothing, and intensity normalization. In raw Raman spectra, different types of noise sources such as offsets, bending and autofluorescence contributions are easily generated. Baseline correction and smoothing remove these noises, while the normalization operation converts all the spectral data into numbers lying between 0 and 1 or −1 and 1, which eliminates the intensity-dependent variations. High-accuracy cancer classification can be achieved in medical diagnosis, especially in research based on the combination of spectral analysis techniques and machine learning techniques^167–175^.

**Fig. 27:**
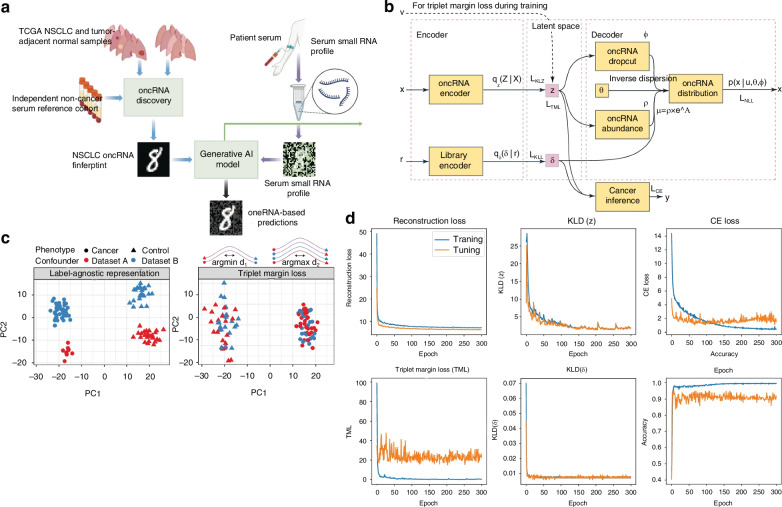
oncRNA-based liquid biopsy platform and Orion architecture^176^. **a** Validation diagram of oncogenic RNAs (oncRNAs) for non-small cell lung cancer (NSCLC) from The Cancer Genome Atlas (TCGA) tissue datasets and non-cancer controls. **b** Schematic diagram of the two required input count matrices for the Orion model architecture. **c** Schematic diagram of the application of triplet margin loss on simulated data. **d** Loss convergence plot. © Springer Nature

As shown in Fig. 27, in the latest study by Goodarzi’s team, a liquid biopsy approach based on a generative AI model (Orion) was proposed to enable the detection of early-stage non-small cell lung cancer (NSCLC) through the analysis of circulating orphan non-coding RNAs (oncRNAs)^176^. The team utilized a variational autoencoder (VAE) to perform multi-task learning on serum samples from 1050 NSCLC patients and matched controls, integrating contrastive learning to eliminate technical confounders (e.g., sequencing depth, sample source variability). Additionally, zero-inflated negative binomial distribution was employed to model the sparse oncRNA expression data. The model achieved an overall sensitivity of 94% and specificity of 87%, with a particularly notable sensitivity of 90% in early-stage detection, significantly outperforming other existing methods.

The integration of machine learning and spectroscopic techniques not only holds promising applications in the field of medical diagnostics but also demonstrates extensive potential in complex multi-component analyses for environmental pollution^177,178^, chemical engineering^179^, and related areas^180–182^. The incorporation of synergy technology effectively mitigates human-induced errors inherent in manual analysis. Through extensive training on large databases, the analytical accuracy can be elevated to over 98%, achieving high-precision analysis in detection domains. Researchers, in addition to developing highly sensitive optofluidic SERS sensing probes, can leverage emerging machine learning technologies to progressively advance SERS spectroscopy into a practical stage characterized by ultra-sensitivity and high precision in detection.

## Conclusion and discussion

Raman spectroscopy has emerged as a cornerstone technique for molecular fingerprinting, yet conventional SERS methods remain constrained by alignment complexity, limited signal collection efficiency, and operational inflexibility^183^. The integration of SERS with optical waveguide architectures, particularly microstructured optical fibers (MOFs)^184^, has revolutionized Raman sensing by addressing these limitations through evanescent field amplification, microfluidic integration, and portable device design. This review systematically categorizes two transformative strategies: (1) SERS-functionalized fiber probes for remote sensing and (2) optofluidic SERS platforms leveraging PCFs. Despite achieving attomolar sensitivity and real-time monitoring, key challenges remain:

Tapered Optical Fibers

- Preparation Complexity: Fabricating tapered fibers and integrating SERS nanoparticles requires precise and intricate processes, making large-scale production and standardization difficult.
- Sample Handling: Tapered fibers often demand a larger volume of sample solution for effective interaction, which may not be practical for trace-level sample analysis.
- Inconsistent Performance: Variability in the tapering process can lead to non-uniform optical and sensing properties across different fibers.

D-type Optical Fibers

1. Limited Durability: The structural modifications to expose larger evanescent fields may compromise the mechanical robustness of the fiber, affecting long-term reliability.
2. Complex Fabrication: Achieving precise structural designs to optimize the evanescent field exposure remains challenging and resource-intensive.
3. Signal Noise and Sensitivity: While D-type fibers provide greater evanescent field interaction, they may suffer from noise interference, reducing the clarity of Raman signals.

Photonic Crystal Optical Fibers (PCFs)

1. Internal Bandgap Constraints: The internal structure of PCFs limits the ability to construct a fully integrated optofluidic Raman sensor. The inability to open the fiber for direct sample interaction hinders practical application in real-time fluidic systems.
2. Sample Compatibility: PCFs often require pre-mixed solutions or specific configurations, which may not align with standard biological or chemical workflows.

These limitations highlight the need for further innovation in optical fiber design to balance sensitivity, robustness, and ease of fabrication while addressing practical requirements for high-throughput and trace-level detection. Recent advancements include the use of microstructured optical fibers (MOFs) and other specialty fibers. In contrast to PCFs, MOFs feature open-channel architectures with integrated microfluidics, enabling real-time interaction between trace samples and SERS substrates within suspended cores. This design prolongs the light-matter interaction length, yielding significantly enhanced detection sensitivity.

The fusion of optical waveguides and SERS has transitioned Raman sensing from a laboratory technique to a transformative tool for precision medicine, environmental stewardship, and industrial automation. While challenges in scalability and reproducibility persist, innovations in novel fabrication techniques for ordered nanomaterials, cavity-enhanced quantum optics, and AI-driven design are poised to unlock single-molecule sensitivity and disposable point-of-care diagnostic platforms. To accelerate translation, future research should focus on:

1. Integrating advanced femtosecond laser lithography to optimize SERS substrates enables the fabrication of uniformly structured and tightly spaced, specially shaped nanoparticles on waveguide surfaces, generating intense “hot spots” that significantly enhance the Raman scattering effect.
2. Innovations in fabrication techniques, such as 3D printing and nanolithography, can reduce the cost of producing optical waveguides and SERS substrates, making the technology accessible for widespread adoption.
3. Combining multi-modal platforms: integrating optical waveguide SERS technology with cavity-enhanced techniques, designing the internal cavity structure to establish a stable standing wave mode for both excitation light and scattered photon signals, thereby enhancing the interaction strength between excitation light and the material and improving light utilization efficiency. Simultaneously, this approach transcends the traditional one-dimensional signal collection mode of optical waveguide SERS probes, enhancing signal collection efficiency. Based on cavity quantum optics theory, this strategy holds the potential to elevate cavity-structured optical waveguide SERS probes into a practical quantitative analysis stage, positioning SERS-based innovations as leaders in the international market.
4. Pairing SERS-integrated optical waveguides with machine learning algorithms can enhance data analysis, enabling real-time identification of complex spectral patterns for accurate diagnostics. AI-driven tools can also improve signal-to-noise ratios by filtering out background interference and standardizing results across different devices.
5. Miniaturizing SERS-integrated optical waveguide systems can lead to highly portable Raman spectrometers suitable for point-of-care testing and field applications.

SERS-integrated optical waveguide systems are poised for widespread adoption, driven by advancements in materials science, nanotechnology, and AI. These technologies hold transformative potential for ultra-sensitive and portable trace liquid analysis across a range of applications. In medical diagnostics, they can facilitate the early detection of diseases like cancer, diabetes, and infectious diseases by analyzing biomarkers in blood, saliva, or urine^185–187^. In environmental monitoring, portable SERS devices can precisely detect pollutants and toxins in water, soil, and air^188,189^. For food safety, they enable trace-level detection of contaminants, pathogens, and pesticides^190–192^. In the chemical industry, they enhance real-time monitoring of industrial processes. By addressing key challenges in these areas, SERS-integrated systems are poised to provide transformative solutions for ultra-sensitive and portable trace liquid analysis, solidifying their role as indispensable tools in 21st-century analytical science.

## References

[1] Dong, S. L. et al. Early cancer detection by serum biomolecular fingerprinting spectroscopy with machine learning. *eLight* **3**, 17 (2023).

[2] Lee, S. et al. Combinatorial effect of tricomponent dual-rim nanoring building blocks: label-free SERS detection of biomolecules. *Nano Lett.* **24**, 3930–3936 (2024). 38513221 10.1021/acs.nanolett.4c00083

[3] Lin, H. & Cheng, J. Computational coherent Raman scattering imaging: breaking physical barriers by fusion of advanced instrumentation and data science. *eLight* **3**, 6 (2023).

[4] Zhang, R. Z. et al. Efficient 2D MOFs nanozyme combining with magnetic SERS substrate for ultrasensitive detection of Hg^2+^. *Spectrochim. Acta Part A Mol. Biomol. Spectrosc.* **312**, 124062 (2024). 10.1016/j.saa.2024.124062 38401506

[5] Lu, X. et al. Single-shot single-beam coherent Raman scattering thermometry based on optically induced air lasing. *Light Sci. Appl.* **13**, 315 (2024). 39582004 10.1038/s41377-024-01598-9 PMC11586427

[6] Lin, S. et al. Time-of-flight resolved stimulated Raman scattering microscopy using counter-propagating ultraslow Bessel light bullets generation. *Light Sci. Appl.* **13**, 148 (2024). 38951517 10.1038/s41377-024-01498-y PMC11217417

[7] Xu, K. et al. Ultrafast laser-induced decomposition for selective activation of GaAs. *Light Adv. Manuf.* **5**, 26 (2024).

[8] Wen, C. C. et al. Surface-enhanced Raman probes based on gold nanomaterials for in vivo diagnosis and imaging. *Chem. Asian J.* **17**, e202200014 (2022). 35178878 10.1002/asia.202200014

[9] Yang, H. et al. Twinned copper nanoparticles modulated with electrochemical deposition for in situ SERS monitoring. *CrystEngComm* **20**, 5609–5618 (2018).

[10] Ankamwar, B. & Sur, U. K. Copper micro/nanostructures as effective SERS active substrates for pathogen detection. *Adv. Nano Res.* **9**, 113–122 (2020).

[11] Wang, S. Y. et al. A surface-enhanced Raman scattering optrode prepared by in situ photoinduced reactions and its application for highly sensitive on-chip detection. *ACS Appl. Mater. Interfaces* **6**, 11706–11713 (2014). 24978908 10.1021/am503881h

[12] Yang, F. et al. Printer-assisted array flexible surface-enhanced Raman spectroscopy chip preparation for rapid and label-free detection of bacteria. *J. Raman Spectrosc.* **51**, 932–940 (2020).

[13] D’Agostino, A. et al. In situ seed-growth synthesis of silver nanoplates on glass for the detection of food contaminants by surface enhanced Raman scattering. *Talanta* **216**, 120936 (2020). 32456888 10.1016/j.talanta.2020.120936

[14] Yang, A. P. et al. Strong interference-resistant flexible fluorescent fiber optic temperature sensor based on fluorescence intensity ratio technology. *Laser Optoelectron. Prog.* **60**, 1316005 (2023).

[15] Wang, Z. et al. Novel optical fiber-based structures for plasmonics sensors. *Biosensors* **12**, 1016 (2022). 36421134 10.3390/bios12111016 PMC9688463

[16] Zhao, J. et al. Review of femtosecond laser direct writing fiber-optic structures based on refractive index modification and their applications. *Opt. Laser Technol.* **146**, 107473 (2022).

[17] Stepniewski, G. et al. From D-shaped to D-shape optical fiber-A universal solution for sensing and biosensing applications: drawn D-shape fiber and its sensing applications. *Measurement* **222**, 113642 (2023).

[18] Xu, W. J. et al. Recent advances in optical fiber high-temperature sensors and encapsulation technique [Invited]. *Chin. Opt. Lett.* **21**, 090007 (2023).

[19] Choudhary, R. et al. Optical fiber axicon enabled plasmofluidic trapping: sensing with enhanced figure of merit. *IEEE Photonics Technol. Lett.* **35**, 469–472 (2023).

[20] Li, Y. J. et al. Difunctional hydrogel optical fiber fluorescence sensor for continuous and simultaneous monitoring of glucose and pH. *Biosensors* **13**, 287 (2023). 36832053 10.3390/bios13020287 PMC9954304

[21] Peng, J. K. et al. Optical fiber temperature and humidity dual parameter sensing based on fiber Bragg gratings and porous film. *Sensors* **23**, 7587 (2023). 37688043 10.3390/s23177587 PMC10490672

[22] Jia, Q. et al. Fibre tapering using plasmonic microheaters and deformation-induced pull. *Light Adv. Manuf.* **4**, 5 (2023).

[23] Panneerselvam, R. et al. Microfluidics and surface-enhanced Raman spectroscopy, a win-win combination? *Lab a Chip* **22**, 665–682 (2022). 10.1039/d1lc01097b 35107464

[24] Bello, J. M. et al. Fiber-optic remote sensor for in situ surface-enhanced Raman scattering analysis. *Anal. Chem.* **62**, 2437–2441 (1990).

[25] Yang, X. et al. In-situ reversible temperature-dependent surface enhanced Raman scattering study using optical fibers. *Chem. Phys. Lett.* **495**, 109–112 (2010).

[26] Toge, K., Hogari, K. & Horiguchi, T. Performance prospects for distributed measurement of Raman gain characteristics in optical fibers. *J. Lightwave Technol.* **22**, 1701–1706 (2004).

[27] Wang, Z. Y. et al. Area-detection fibre-optic system for spatially offset Raman spectroscopy and Raman tomography in reflection mode. *Electron. Lett.* **51**, 1684–1686 (2015).

[28] Wang, C. et al. Review of optical fibre probes for enhanced Raman sensing. *J. Raman Spectrosc.* **48**, 1040–1055 (2017).

[29] Liu, Y. et al. Stick-slip-motion-assisted interfacial self-assembly of noble metal nanoparticles on tapered optical fiber surface and its application in SERS detection. *Appl. Surf. Sci.* **602**, 154298 (2022).

[30] Taguenang, J. M. et al. Surface enhanced Raman spectroscopy on the tip of a plastic optical fiber. *Proceedings of SPIE 6641, Plasmonics: Metallic Nanostructures and Their Optical Properties V*. 66411X (SPIE, 2007).

[31] Viets, C. & Hill, W. Single-fibre surface-enhanced Raman sensors with angled tips. *J. Raman Spectrosc.* **31**, 625–631 (2000).

[32] Viets, C. & Hill, W. Fibre-optic SERS sensors with angled tips. *J. Mol. Struct.* **565-566**, 515–518 (2001).

[33] Pisco, M. et al. Reproducible SERS substrates on optical fiber tips by nanosphere lithography. *2017 25th Optical Fiber Sensors Conference (OFS)*. 1-4 (IEEE, 2017).

[34] Meng, L. P. et al. Optical fiber optrodes with silver-coated gold nanocavity ordered arrays for highly sensitive surface enhanced Raman spectrum. *Sens. Actuators B: Chem.* **380**, 133314 (2023).

[35] Stokes, D. L., Chi, Z. H. & Vo-Dinh, T. Surface-enhanced-Raman-scattering-inducing nanoprobe for spectrochemical analysis. *Appl. Spectrosc.* **58**, 292–298 (2004). 15035709 10.1366/000370204322886636

[36] Kim, J. A. et al. Fiber-optic SERS probes fabricated using two-photon polymerization for rapid detection of bacteria. *Adv. Opt. Mater.* **8**, 1901934 (2020).

[37] Huang, R. et al. High-sensitivity and throughput optical fiber SERS probes based on laser-induced fractional reaction method. *Results Phys.* **48**, 106410 (2023).

[38] Gu, C. et al. Highly sensitive and compact molecular sensor using surface enhanced Raman scattering and optical fibers. *2007 Conference on Lasers and Electro-Optics - Pacific Rim*. 1-2 (IEEE, 2007).

[39] Schmidt, H. & Hawkins, A. R. Optofluidic waveguides: I. concepts and implementations. *Microfluidics Nanofluidics* **4**, 3–16 (2008). 21442048 10.1007/s10404-007-0199-7 PMC3062956

[40] Birks, T. A. et al. Full 2-D photonic bandgaps in silica/air structures. *Electron. Lett.* **31**, 1941–1943 (1995).

[41] Knight, J. C. et al. All-silica single-mode optical fiber with photonic crystal cladding. *Opt. Lett.* **21**, 1547–1549 (1996). 19881720 10.1364/ol.21.001547

[42] Cregan, R. F. et al. Single-mode photonic band gap guidance of light in air. *Science* **285**, 1537–1539 (1999). 10477511 10.1126/science.285.5433.1537

[43] Fard, A. P. et al. The design of a photonic crystal fiber for hydrogen cyanide gas detection. *Photonics* **11**, 178 (2024).

[44] Gomółka, G. et al. Dual-pass hollow-core fiber gas spectroscopy using a reflective configuration with heterodyne-based signal detection. *J. Lightwave Technol.* **41**, 6094–6101 (2023).

[45] Alam, M. K. et al. RETRACTED ARTICLE: design of highly sensitive biosensors using hollow-core microstructured fibers for plasma sensing in aids with human metabolism. *Opt. Quantum Electron.* **55**, 188 (2023). 36618531 10.1007/s11082-022-04514-w PMC9811872

[46] Gimbert, L. J. & Worsfold, P. J. Environmental applications of liquid-waveguide-capillary cells coupled with spectroscopic detection. *TrAC Trends Anal. Chem.* **26**, 914–930 (2007).

[47] Yuan, W. H. et al. A ring core fiber sensor based on Mach-Zehnder interferometer for transversal force sensing with solvable temperature cross sensitivity. *IEEE Sens. J.* **23**, 3615–3622 (2023).

[48] Saitoh, K. et al. Chromatic dispersion control in photonic crystal fibers: application to ultra-flattened dispersion. *Opt. Express* **11**, 843–852 (2003). 19461798 10.1364/oe.11.000843

[49] Paget, B. M. et al. A review on photonic crystal fiber based fluorescence sensing for chemical and biomedical applications. *Sens. Actuators B Chem.* **400**, 134828 (2024).

[50] Tao, Y. et al. Refractive index sensing simulations of CsPbBr_3_ quantum dots/gold bilayer coated triangular-lattice photonic crystal fibers. *Photonic Sens.* **12**, 220309 (2022).

[51] Corbett, J. et al. The coupling performance of photonic crystal fibres in fibre stellar interferometry. *Monthly Not. R. Astronomical Soc.* **368**, 203–210 (2006).

[52] Zhou, X. et al. The rise of graphene photonic crystal fibers. *Adv. Funct. Mater.* **32**, 2202282 (2022).

[53] Fan, Z. K. et al. Two kinds of liquid crystal filled PCFs temperature and RI sensors based on SPR. *IEEE Sens. J.* **23**, 5766–5772 (2023).

[54] Wang, J. X. et al. Ultra-high sensitivity photonic crystal fiber sensor based on dispersion turning point sensitization of surface plasmonic polariton modes for low RI liquid detection. *Opt. Express* **32**, 32895–32908 (2024). 39573004 10.1364/OE.531112

[55] Sun, Z. T. et al. Surface mode enhanced by avoided crossing in microstructure fibers for improved SERS sensing. *Sens. Actuators B: Chem.* **368**, 132249 (2022).

[56] Han, Y. et al. Towards full-length accumulative surface-enhanced Raman scattering-active photonic crystal fibers. *Adv. Mater.* **22**, 2647–2651 (2010). 20440699 10.1002/adma.200904192

[57] Khetani, A. et al. Hollow core photonic crystal fiber for monitoring leukemia cells using surface enhanced Raman scattering (SERS). *Biomed. Opt. Express* **6**, 4599–4609 (2015). 26601021 10.1364/BOE.6.004599 PMC4646565

[58] Hanf, S. et al. Fiber-enhanced Raman multigas spectroscopy: a versatile tool for environmental gas sensing and breath analysis. *Anal. Chem.* **86**, 5278–5285 (2014). 24846710 10.1021/ac404162w

[59] Hanf, S. et al. Fast and highly sensitive fiber-enhanced Raman spectroscopic monitoring of molecular H_2_ and CH_4_ for point-of-care diagnosis of malabsorption disorders in exhaled human breath. *Anal. Chem.* **87**, 982–988 (2015). 25545503 10.1021/ac503450y

[60] Shi, C. et al. Inner wall coated hollow core waveguide sensor based on double substrate surface enhanced Raman scattering. *Appl. Phys. Lett.* **93**, 153101 (2008).

[61] Olson, T. Y. et al. Raman and surface-enhanced Raman detection of domoic acid and saxitoxin. *Appl. Spectrosc.* **65**, 159–164 (2011).

[62] Benazza, A. et al. Reliable and easy-to-use SERS spectroscopy probe using a tapered opto-fluidic photonic crystal fiber. *Opt. Express* **32**, 3440–3450 (2024). 38297564 10.1364/OE.501911

[63] Fan, X. D. & White, I. M. Optofluidic microsystems for chemical and biological analysis. *Nat. Photonics* **5**, 591–597 (2011). 22059090 10.1038/nphoton.2011.206 PMC3207487

[64] Wang, J. et al. AgCuInCdZn high-entropy alloy nanoparticles-embedded in porous carbon fibers for long-cycling lithium metal anodes. *Chem. Eng. J.* **477**, 146884 (2023).

[65] Gorai, P. et al. Molecular imprinting polymer nanoparticles coupled with an optical sensor for sensitive and label-free detection of *p*-cresol. *ACS Appl. Nano Mater.* **6**, 12946–12956 (2023).

[66] Jha, R. et al. Label-free biochemical sensing using processed optical fiber interferometry: a review. *ACS Omega* **9**, 3037–3069 (2024). 38284054 10.1021/acsomega.3c03970 PMC10809379

[67] Beffara, F. et al. Development of highly reliable SERS-active photonic crystal fiber probe and its application in the detection of ovarian cancer biomarker in cyst fluid. *J. Biophotonics* **13**, e201960120 (2020). 31814313 10.1002/jbio.201960120

[68] Dinish, U. S. et al. Surface-enhanced Raman scattering-active photonic crystal fiber probe: towards next generation liquid biopsy sensor with ultra high sensitivity. *J. Biophotonics* **12**, e201900027 (2019). 30891937 10.1002/jbio.201900027

[69] Beffara, F. et al. Optimization and performance analysis of SERS-active suspended core photonic crystal fibers. *Opt. Express* **28**, 23609–23619 (2020). 32752354 10.1364/OE.393251

[70] Humbert, G. et al. Hollow core photonic crystal fibers for beam delivery. *Opt. Express* **12**, 1477–1484 (2004). 19474973 10.1364/opex.12.001477

[71] Knight, J. C. et al. Photonic band gap guidance in optical fibers. *Science* **282**, 1476–1478 (1998). 9822375 10.1126/science.282.5393.1476

[72] Bennett, P. J., Monro, T. M. & Richardson, D. J. Toward practical holey fiber technology: fabrication, splicing, modeling, and characterization. *Opt. Lett.* **24**, 1203–1205 (1999). 18073984 10.1364/ol.24.001203

[73] Pustelny, T. & Grabka, M. Photonic-crystal fibres with suspended core-Numerical analyses. *Acta Phys. Polonica A* **114**, A-115–A-120 (2008).

[74] Euser, T. G. et al. Dynamic control of higher-order modes in hollow-core photonic crystal fibers. *Opt. Express* **16**, 17972–17981 (2008). 18958077 10.1364/oe.16.017972

[75] Markin, A. V., Markina, N. E. & Goryacheva, I. Y. Raman spectroscopy based analysis inside photonic-crystal fibers. *TrAC Trends Anal. Chem.* **88**, 185–197 (2017).

[76] Bratashov, D. N. et al. Microstructured waveguides with polyelectrolyte-stabilized gold nanostars for SERS sensing of dissolved analytes. *Materials* **11**, 734 (2018). 29734729 10.3390/ma11050734 PMC5978111

[77] Skibina, Y. S. et al. Photonic crystal fibres in biomedical investigations. *Quantum Electron.* **41**, 284–301 (2011).

[78] Merdalimova, A. A. et al. SERS platform based on hollow-core microstructured optical fiber: technology of UV-mediated gold nanoparticle growth. *Biosensors* **12**, 19 (2022). 10.3390/bios12010019 PMC8774134 35049647

[79] Chen, X. et al. High-fidelity, low-latency polarization quantum state transmissions over a hollow-core conjoined-tube fiber at around 800 nm. *Photonics Res.* **9**, 460 (2021).

[80] Hong, Y. F. et al. Highly birefringent anti-resonant hollow-core fiber with a Bi-thickness fourfold semi-tube structure. *Laser Photonics Rev.* **16**, 2100365 (2022).

[81] Taranta, A. et al. Exceptional polarization purity in antiresonant hollow-core optical fibres. *Nat. Photonics* **14**, 504–510 (2020).

[82] Xia, Z. W. et al. Giant enhancement of Raman scattering by a hollow-core microstructured optical fiber allows single exosome probing. *ACS Sens.* **8**, 1799–1809 (2023). 37018734 10.1021/acssensors.3c00131

[83] Russell, P. Photonic crystal fibers. *Science* **299**, 358–362 (2003). 12532007 10.1126/science.1079280

[84] Warren-Smith, S. C. et al. Exposed-core microstructured optical fibers for real-time fluorescence sensing. *Opt. Express* **17**, 18533–18542 (2009). 20372584 10.1364/OE.17.018533

[85] Coscelli, E. et al. Toward a highly specific DNA biosensor: PNA-modified suspended-core photonic crystal fibers. *IEEE J. Sel. Top. Quantum Electron.* **16**, 967–972 (2010).

[86] Zhang, T. et al. Design and fabrication of a novel core-suspended optic fiber for distributed gas sensor. *Proceedings of SPIE 8924, Fourth Asia Pacific Optical Sensors Conference*. 892402 (SPIE, 2013).

[87] Li, X. G. et al. Label free optofluidic DNA hybridization detection based on suspended core fiber whispering gallery mode resonator. *Measurement* **222**, 113661 (2023).

[88] Xue, J. et al. Ultra-high sensitivity terahertz microstructured fiber biosensor for diabetes mellitus and coronary heart disease marker detection. *Sensors* **23**, 2020 (2023). 36850616 10.3390/s23042020 PMC9962755

[89] Heng, S. et al. Microstructured optical fiber-based biosensors: reversible and nanoliter-scale measurement of zinc ions. *ACS Appl. Mater. Interfaces* **8**, 12727–12732 (2016). 27152578 10.1021/acsami.6b03565

[90] Kassani, S. H. et al. Suspended ring-core photonic crystal fiber gas sensor with high sensitivity and fast response. *IEEE Photonics J.* **7**, 2700409 (2015).

[91] Liu, L. L. et al. Chemically functionalised suspended-core fibre for ammonia gas detection. *J. Lightwave Technol.* **39**, 5197–5205 (2021).

[92] Paixão, T. et al. Fabry-Perot interferometer based on suspended core fiber for detection of gaseous ethanol. *Appl. Sci.* **12**, 726 (2022).

[93] Menzel, E. R., Menzel, L. W. & Schwierking, J. R. A photoluminescence-based field method for detection of traces of explosives. *Sci. World J.* **4**, 725–735 (2004). 10.1100/tsw.2004.126 PMC5956434 15349512

[94] Kneipp, K. et al. Near-infrared surface-enhanced Raman scattering of trinitrotoluene on colloidal gold and silver. *Spectrochim. Acta Part A: Mol. Biomol. Spectrosc.* **51**, 2171–2175 (1995).

[95] Lewis, M. L., Lewis, I. R. & Griffiths, P. R. Anti-stokes Raman spectrometry with 1064-nm excitation: an effective instrumental approach for field detection of explosives. *Appl. Spectrosc.* **58**, 420–427 (2004). 17140491 10.1366/000370204773580266

[96] Sylvia, J. M. et al. Surface-enhanced Raman detection of 2,4-dinitrotoluene impurity vapor as a marker to locate landmines. *Anal. Chem.* **72**, 5834–5840 (2000). 11128944 10.1021/ac0006573

[97] Fang, X. & Ahmad, S. R. Detection of explosive vapour using surface-enhanced Raman spectroscopy. *Appl. Phys. B* **97**, 723–726 (2009).

[98] Monro, T. M. et al. Sensing with suspended-core optical fibers. *Opt. Fiber Technol.* **16**, 343–356 (2010).

[99] Zhu, Y. N., Du, H. & Bise, R. Design of solid-core microstructured optical fiber with steering-wheel air cladding for optimal evanescent-field sensing. *Opt. Express* **14**, 3541–3546 (2006). 19516500 10.1364/oe.14.003541

[100] Wang, G. J. et al. Gas Raman sensing with multi-opened-up suspended core fiber. *Appl. Opt.* **50**, 6026–6032 (2011). 22086030 10.1364/AO.50.006026

[101] Xu, Y. Y. et al. Surface-enhanced Raman scattering in silver-coated suspended-core fiber. *Sensors* **24**, 160 (2024). 10.3390/s24010160 PMC10781242 38203021

[102] Gao, D. H. et al. Optofluidic in-fiber integrated surface-enhanced Raman spectroscopy detection based on a hollow optical fiber with a suspended core. *Opt. Lett.* **44**, 5173–5176 (2019). 31674959 10.1364/OL.44.005173

[103] Oo, M. K. K. et al. Structure fits the purpose: photonic crystal fibers for evanescent-field surface-enhanced Raman spectroscopy. *Opt. Lett.* **35**, 466–468 (2010). 20160786 10.1364/OL.35.000466

[104] Teng, P. P. et al. In situ SERS detection of quinolone antibiotic residues in a water environment based on optofluidic in-fiber integrated Ag nanoparticles. *Appl. Opt.* **60**, 6659–6664 (2021). 34612910 10.1364/AO.426611

[105] Gao, D. H. et al. Surface-enhanced Raman spectroscopy detection of cerebrospinal fluid glucose based on the optofluidic in-fiber-integrated composites of graphene oxide, silver nanoparticles, and 4-mercaptophenylboronic acid. *ACS Appl. Nano Mater.* **4**, 10784–10790 (2021).

[106] Gao, D. H. et al. On-line SERS detection of adenine in DNA based on the optofluidic in-fiber integrated GO/PDDA/Ag NPs. *Sens. Actuators B Chem.* **332**, 129517 (2021).

[107] Gao, D. H. et al. On-line SERS detection of bilirubin based on the optofluidic in-fiber integrated GO/Ag NPs for rapid diagnosis of jaundice. *Talanta* **234**, 122692 (2021). 34364489 10.1016/j.talanta.2021.122692

[108] Sun, Z. T. et al. Plasmonic Ag/ZnO nanoscale villi in microstructure fibers for sensitive and reusable surface-enhanced Raman scattering sensing. *ACS Appl. Nano Mater.* **6**, 714–719 (2023).

[109] Schroder, K. et al. Functionalization of microstructured optical fibers by internal nanoparticle mono-layers for plasmonic biosensor applications. *IEEE Sens. J.* **12**, 218–224 (2012).

[110] Zhao, Y. Q. et al. Plasmonic nanopillar array embedded microfluidic chips: an in situ SERS monitoring platform. *J. Mater. Chem. A* **3**, 6408–6413 (2015).

[111] Duan, T. L. et al. A novel fabrication technique for high-aspect-ratio nanopillar arrays for SERS application. *RSC Adv.* **10**, 45037–45041 (2020). 35516272 10.1039/d0ra09145f PMC9058654

[112] Gutierrez-Rivera, L. et al. Application of EBL fabricated nanostructured substrates for surface enhanced Raman spectroscopy detection of protein A in aqueous solution. *J. Vac. ScienceTechnology B* **31**, 06F901 (2013).

[113] Peters, R. F. et al. Surface enhanced Raman spectroscopy detection of biomolecules using EBL fabricated nanostructured substrates. *J. Visual. Exp.* **97**, e52712 (2015). 10.3791/52712 PMC4401373 25867853

[114] Cinel, N. A. et al. E-Beam lithography designed substrates for surface enhanced Raman spectroscopy. *Photonics Nanostruct. Fundamentals Appl.* **15**, 109–115 (2015).

[115] Abu Hatab, N. A., Oran, J. M. & Sepaniak, M. J. Surface-enhanced Raman spectroscopy substrates created *via* electron beam lithography and nanotransfer printing. *ACS Nano* **2**, 377–385 (2008). 19206640 10.1021/nn7003487

[116] Lin, Y. Y. et al. Focused ion beam-fabricated Au micro/nanostructures used as a surface enhanced Raman scattering-active substrate for trace detection of molecules and influenza virus. *Nanotechnology* **22**, 185308 (2011). 21427472 10.1088/0957-4484/22/18/185308

[117] Gao, T. T. et al. High performance surface-enhanced Raman scattering substrates of Si-based Au film developed by focused ion beam nanofabrication. *Nanoscale Res. Lett.* **7**, 399 (2012). 22804810 10.1186/1556-276X-7-399 PMC3502558

[118] Dhawan, A., Gerhold, M. & Vo-Dinh, T. Theoretical simulation and focused ion beam fabrication of gold nanostructures for surface-enhanced Raman scattering (SERS). *NanoBiotechnology* **3**, 164–171 (2007). 23976888 10.1007/s12030-008-9017-x PMC3748982

[119] Sivashanmugan, K. et al. Focused-ion-beam-fabricated Au/Ag multilayered nanorod array as SERS-active substrate for virus strain detection. *Sens. Actuators B: Chem.* **181**, 361–367 (2013).

[120] Prakash, J. et al. Ion beam nanoengineering of surfaces for molecular detection using surface enhanced Raman scattering. *Mol. Syst. Des. Eng.* **7**, 411–421 (2022).

[121] Zhang, Y. L. et al. Designable 3D nanofabrication by femtosecond laser direct writing. *Nano Today* **5**, 435–448 (2010).

[122] Zacharatos, F. et al. Laser printing of Au nanoparticles with sub-micron resolution for the fabrication of monochromatic reflectors on stretchable substrates. *Opt. Laser Technol.* **135**, 106660 (2021).

[123] Zhou, J. et al. Rapid selective ablation and high-precision patterning for micro-thermoelectric devices using femtosecond laser directing writing. *ACS Appl. Mater. Interfaces* **14**, 3066–3075 (2022). 34985853 10.1021/acsami.1c21326

[124] Gissibl, T. et al. Two-photon direct laser writing of ultracompact multi-lens objectives. *Nat. Photonics* **10**, 554–560 (2016).

[125] Tenbrake, L. et al. Direct laser-written optomechanical membranes in fiber Fabry-Perot cavities. *Nat. Commun.* **15**, 209 (2024). 38172102 10.1038/s41467-023-44490-7 PMC10764917

[126] Li, X. Y. et al. A self-driven microfluidic surface-enhanced Raman scattering device for Hg^2+^ detection fabricated by femtosecond laser. *Lab a Chip* **20**, 414–423 (2020). 10.1039/c9lc00883g 31867593

[127] Zhou, T. H. et al. Superhydrophobic/-philic SERS platform based on femtosecond laser-induced periodic surface structures and Ag nanoparticles. *ACS Appl. Nano Mater.* **7**, 25014–25024 (2024).

[128] Bai, S. et al. λ/20 surface nanostructuring of ZnO by mask-less ultrafast laser processing. *Nanophotonics* **12**, 1499–1510 (2023). 39634593 10.1515/nanoph-2022-0657 PMC11501954

[129] Premachandran, S. et al. Fabrication of isotope-enriched nanostructures using ultrafast laser pulses under ambient conditions for biomolecular sensing. *Adv. Mater.* **36**, 2406081 (2024). 10.1002/adma.202406081 38886842

[130] Xu, L. M. et al. Fabrication of SERS substrates by femtosecond LIPAA for detection of contaminants in foods. *Opt. Laser Technol.* **151**, 107954 (2022).

[131] Zhang, Y. F. et al. Femtosecond laser fabrication of nanopillar arrays for surface-enhanced Raman scattering substrates. *Opt. Laser Technol.* **181**, 111659 (2025).

[132] Xu, Y. W. et al. Femtosecond laser ablated pyramidal fiber taper-SERS probe with laser-induced silver nanostructures. *J. Phys. D: Appl. Phys.* **51**, 285104 (2018).

[133] Hsu, Y. T. et al. Femtosecond laser-induced Au nanostructure-decorated with plasmonic nanomaterials for sensitive SERS-based detection of fentanyl. *Talanta* **284**, 127264 (2025). 39581107 10.1016/j.talanta.2024.127264

[134] Hu, Y. X. et al. Femtosecond laser-induced nanoparticle implantation into flexible substrate for sensitive and reusable microfluidics SERS detection. *Int. J. Extrem. Manuf.* **6**, 045005 (2024).

[135] Jahani, S. & Jacob, Z. All-dielectric metamaterials. *Nat. Nanotechnol.* **11**, 23–36 (2016). 26740041 10.1038/nnano.2015.304

[136] Huang, S. H. et al. Surface-enhanced Raman scattering on dielectric microspheres with whispering gallery mode resonance. *Photonics Res.* **6**, 346–356 (2018).

[137] Das, G. M. et al. Numerical investigations on photonic nanojet mediated surface enhanced Raman scattering and fluorescence techniques. *Opt. Express* **25**, 19822–19831 (2017). 29041669 10.1364/OE.25.019822

[138] Wang, M. Y. et al. Flexible microsphere-coupled surface-enhanced Raman spectroscopy (McSERS) by dielectric microsphere cavity array with random plasmonic nanoparticles. *J. Raman Spectrosc.* **53**, 1238–1248 (2022).

[139] Lin, S. Y. et al. Surface-enhanced Raman scattering with Ag Nanoparticles optically trapped by a photonic crystal cavity. *Nano Lett.* **13**, 559–563 (2013). 23339834 10.1021/nl304069n

[140] Jiang, X. F. et al. Whispering-gallery sensors. *Matter* **3**, 371–392 (2020). 32835223 10.1016/j.matt.2020.07.008 PMC7405776

[141] Yu, D. S. et al. Whispering-gallery-mode sensors for biological and physical sensing. *Nat. Rev. Methods Prim.* **1**, 83 (2021).

[142] He, L. N., Özdemir, Ş. K. & Yang, L. Whispering gallery microcavity lasers. *Laser Photonics Rev.* **7**, 60–82 (2013).

[143] Toropov, N. et al. Review of biosensing with whispering-gallery mode lasers. *Light Sci. Appl.* **10**, 42 (2021). 33637696 10.1038/s41377-021-00471-3 PMC7910454

[144] Lin, G. P., Coillet, A. & Chembo, Y. K. Nonlinear photonics with high-*Q* whispering-gallery-mode resonators. *Adv. Opt. Photonics* **9**, 828–890 (2017).

[145] Dantham, V. R. et al. Label-free detection of single protein using a nanoplasmonic-photonic hybrid microcavity. *Nano Lett.* **13**, 3347–3351 (2013). 23777440 10.1021/nl401633y

[146] Baaske, M. D., Foreman, M. R. & Vollmer, F. Single-molecule nucleic acid interactions monitored on a label-free microcavity biosensor platform. *Nat. Nanotechnol.* **9**, 933–939 (2014). 25173831 10.1038/nnano.2014.180

[147] Baaske, M. D. & Vollmer, F. Optical observation of single atomic ions interacting with plasmonic nanorods in aqueous solution. *Nat. Photonics* **10**, 733–739 (2016).

[148] Yu, X. C. et al. Single-molecule optofluidic microsensor with interface whispering gallery modes. *Proc. Natl. Acad. Sci. USA* **119**, e2108678119 (2022). 35115398 10.1073/pnas.2108678119 PMC8832994

[149] L, M. J., Pillanagrovi, J. & Dutta-Gupta, S. Tailoring cavity coupled plasmonic substrates for SERS applications. *Nanotechnology* **34**, 335501 (2023). 10.1088/1361-6528/acd4c7 37172574

[150] Wang, W. A. et al. Protein aggregation monitoring in microdisk optofluidic sensor through microcavity enhanced Raman scattering. *Colloids Surf. A Physicochem. Eng. Asp.* **679**, 132561 (2023).

[151] Ouyang, X. et al. Ultrasensitive optofluidic enzyme-linked immunosorbent assay by on-chip integrated polymer whispering-gallery-mode microlaser sensors. *Lab a Chip* **20**, 2438–2446 (2020). 10.1039/d0lc00240b 32484485

[152] Zhang, J. et al. Whispering-gallery nanocavity plasmon-enhanced Raman spectroscopy. *Sci. Rep.* **5**, 15012 (2015). 26443526 10.1038/srep15012 PMC4595732

[153] Fan, X. C. et al. High-specificity molecular sensing on an individual whispering-gallery-mode cavity: coupling-enhanced Raman scattering by photoinduced charge transfer and cavity effects. *Nanoscale Horiz.* **8**, 195–201 (2023). 36468209 10.1039/d2nh00450j

[154] Mao, W. B. et al. A whispering-gallery scanning microprobe for Raman spectroscopy and imaging. *Light Sci. Appl.* **12**, 247 (2023). 37798286 10.1038/s41377-023-01276-2 PMC10556008

[155] Feng, L. et al. Silver-coated elevated bowtie nanoantenna arrays: improving the near-field enhancement of gap cavities for highly active surface-enhanced raman scattering. *Nano Res.* **8**, 3715–3724 (2015).

[156] Pandey, A. et al. Silica nanospheres coated silver islands as an effective opto-plasmonic SERS active platform for rapid and sensitive detection of prostate cancer biomarkers. *Molecules* **27**, 7821 (2022). 36431921 10.3390/molecules27227821 PMC9697738

[157] Caligiuri, V., Nucera, A., Patra, A., Castriota, M. & Luca, A. D. Raman scattering enhancement through pseudo-cavity modes. *Nanomaterials* **14**, 875 (2024). 38786831 10.3390/nano14100875 PMC11124054

[158] Shlesinger, I. et al. Hybrid cavity-antenna architecture for strong and tunable sideband-selective molecular Raman scattering enhancement. *Sci. Adv.* **9**, eadj4637 (2023). 38117880 10.1126/sciadv.adj4637 PMC10732519

[159] Kim, T. et al. Fabry-Perot cavity control for tunable Raman scattering. *Small* **19**, 2207003 (2023). 10.1002/smll.202207003 37017491

[160] Wang, Z. K. et al. Raman enhancement mechanism and experiments of cavity-enhanced AgNP decorated tapered fiber sensor. *Opt. Lett.* **46**, 4300–4303 (2021). 34469999 10.1364/OL.435839

[161] Cutshaw, G. et al. The emerging role of Raman spectroscopy as an omics approach for metabolic profiling and biomarker detection toward precision medicine. *Chem. Rev.* **123**, 8297–8346 (2023). 37318957 10.1021/acs.chemrev.2c00897 PMC10626597

[162] Fan, X. Q. et al. Deep learning-based component identification for the Raman spectra of mixtures. *Analyst* **144**, 1789–1798 (2019). 30672931 10.1039/c8an02212g

[163] Maleki, F. et al. Machine learning algorithm validation: from essentials to advanced applications and implications for regulatory certification and deployment. *Neuroimaging Clin. North Am.* **30**, 433–445 (2020). 10.1016/j.nic.2020.08.004 33038994

[164] Wang, M. et al. DeFine: deep convolutional neural networks accurately quantify intensities of transcription factor-DNA binding and facilitate evaluation of functional non-coding variants. *Nucleic Acids Res.* **46**, e69 (2018). 29617928 10.1093/nar/gky215 PMC6009584

[165] Rahman, M. A., Zhang, T. J. & Lu, Y. PINN-CHK: physics-informed neural network for high-fidelity prediction of early-age cement hydration kinetics. *Neural Comput. Appl.* **36**, 13665–13687 (2024).

[166] Kondepudi, A. et al. Foundation models for fast, label-free detection of glioma infiltration. *Nature* **637**, 439–445 (2025). 39537921 10.1038/s41586-024-08169-3 PMC11711092

[167] Yang, Y. et al. Human ACE2-functionalized gold “virus-trap” nanostructures for accurate capture of SARS-CoV-2 and single-virus SERS detection. *Nano-Micro Lett.* **13**, 109 (2021). 10.1007/s40820-021-00620-8 PMC8042470 33868761

[168] Zhang, Z. et al. Rapid detection of viruses: based on silver nanoparticles modified with bromine ions and acetonitrile. *Chem. Eng. J.* **438**, 135589 (2022). 35261557 10.1016/j.cej.2022.135589 PMC8890791

[169] Peng, Y. et al. Identifying infectiousness of SARS-CoV-2 by ultra-sensitive SnS_2_ SERS biosensors with capillary effect. *Matter* **5**, 694–709 (2022). 34957388 10.1016/j.matt.2021.11.028 PMC8686209

[170] Ember, K. J. et al. Saliva-based detection of COVID-19 infection in a real-world setting using reagent-free Raman spectroscopy and machine learning. *J. Biomed. Opt.* **27**, 025002 (2022). 35142113 10.1117/1.JBO.27.2.025002 PMC8825664

[171] Chen, Z. R. et al. Physics-informed machine learning enabled virtual experimentation for 3D printed thermoplastic. *Mater. Horiz.* **11**, 6028–6039 (2024). 39356177 10.1039/d4mh01022a

[172] Zhou, H. et al. Machine learning-augmented surface-enhanced spectroscopy toward next-generation molecular diagnostics. *Nanoscale Adv.* **5**, 538–570 (2023). 36756499 10.1039/d2na00608a PMC9890940

[173] Huang, L. P. et al. Rapid, label-free histopathological diagnosis of liver cancer based on Raman spectroscopy and deep learning. *Nat. Commun.* **14**, 48 (2023). 36599851 10.1038/s41467-022-35696-2 PMC9813224

[174] Zhao, X. et al. Plasmonic trimers designed as SERS-active chemical traps for subtyping of lung tumors. *Nat. Commun.* **15**, 5855 (2024). 38997298 10.1038/s41467-024-50321-0 PMC11245553

[175] Richter, T. et al. Delineating the effective use of self-supervised learning in single-cell genomics. *Nat. Mach. Intell.* **7**, 68–78 (2025).

[176] Karimzadeh, M. et al. Deep generative AI models analyzing circulating orphan non-coding RNAs enable detection of early-stage lung cancer. *Nat. Commun.* **15**, 10090 (2024). 39572521 10.1038/s41467-024-53851-9 PMC11582319

[177] Li, Y. L. et al. Tracing microplastic aging processes using multimodal deep learning: a predictive model for enhanced traceability. *Environ. Sci. Technol.* **58**, 18335–18344 (2024). 39251361 10.1021/acs.est.4c05022

[178] Ma, H. Y. et al. Prediction of multilayer Cr/GLC coatings degradation in deep-sea environments based on integrated mechanistic and machine learning models. *Corros. Sci.* **224**, 111513 (2023).

[179] Theobald, N. et al. Identification of unknown nanofabrication chemicals using Raman spectroscopy and deep learning. *IEEE Sens. J.* **23**, 7910–7916 (2023).

[180] Zhou, L. et al. Powdery food identification using NIR spectroscopy and extensible deep learning model. *Food Bioprocess Technol.* **15**, 2354–2362 (2022).

[181] Sun, J. J. et al. Rapid identification of drug mechanisms with deep learning-based multichannel surface-enhanced Raman spectroscopy. *ACS Sens.* **9**, 4227–4235 (2024). 39138903 10.1021/acssensors.4c01205

[182] Zhu, L. J. et al. Open-set deep learning-enabled single-cell Raman spectroscopy for rapid identification of airborne pathogens in real-world environments. *Sci. Adv.* **11**, eadp7991 (2025). 39772685 10.1126/sciadv.adp7991 PMC11708874

[183] Bai, S. & Sugioka, K. Recent advances in the fabrication of highly sensitive surface-enhanced Raman scattering substrates: nanomolar to attomolar level sensing. *Light Adv. Manuf.* **2**, 13 (2021).

[184] Lühder, T. et al. Longitudinally thickness-controlled nanofilms on exposed core fibres enabling spectrally flattened supercontinuum generation. *Light Adv. Manuf.* **2**, 262–273 (2021).

[185] Lee, W. et al. Spread spectrum SERS allows label-free detection of attomolar neurotransmitters. *Nat. Commun.* **12**, 159 (2021). 33420035 10.1038/s41467-020-20413-8 PMC7794485

[186] Shin, H. et al. Single test-based diagnosis of multiple cancer types using Exosome-SERS-AI for early stage cancers. *Nat. Commun.* **14**, 1644 (2023). 36964142 10.1038/s41467-023-37403-1 PMC10039041

[187] Hanna, K. et al. Raman spectroscopy: current applications in breast cancer diagnosis, challenges and future prospects. *Br. J. Cancer* **126**, 1125–1139 (2022). 34893761 10.1038/s41416-021-01659-5 PMC8661339

[188] Guselnikova, O. et al. Pretreatment-free SERS sensing of microplastics using a self-attention-based neural network on hierarchically porous Ag foams. *Nat. Commun.* **15**, 4351 (2024). 38806498 10.1038/s41467-024-48148-w PMC11133413

[189] Yang, S. et al. Monitoring the charge-transfer process in a Nd-doped semiconductor based on photoluminescence and SERS technology. *Light Sci. Appl.* **9**, 117 (2020). 32685138 10.1038/s41377-020-00361-0 PMC7351777

[190] Logan, N. et al. Handheld SERS coupled with QuEChERs for the sensitive analysis of multiple pesticides in basmati rice. *npj Sci. Food* **6**, 3 (2022). 35027565 10.1038/s41538-021-00117-z PMC8758682

[191] Bi, X. Y. et al. Digital colloid-enhanced Raman spectroscopy by single-molecule counting. *Nature* **628**, 771–775 (2024). 38632399 10.1038/s41586-024-07218-1

[192] Zhang, Y. R. et al. General approach to surface-accessible plasmonic Pickering emulsions for SERS sensing and interfacial catalysis. *Nat. Commun.* **14**, 1392 (2023). 36914627 10.1038/s41467-023-37001-1 PMC10011407

